# FusionPathway: Prediction of pathways and therapeutic targets associated with gene fusions in cancer

**DOI:** 10.1371/journal.pcbi.1006266

**Published:** 2018-07-24

**Authors:** Chia-Chin Wu, Hannah C. Beird, Jianhua Zhang, P. Andrew Futreal

**Affiliations:** Department of Genomic Medicine, The University of Texas MD Anderson Cancer Center, Houston, Texas, United States of America; Center for Cancer Research, UNITED KINGDOM

## Abstract

Numerous gene fusions have been uncovered across multiple cancer types. Although the ability to target several of these fusions has led to the development of some successful anti-cancer drugs, most of them are not druggable. Understanding the molecular pathways of a fusion is important in determining its function in oncogenesis and in developing therapeutic strategies for patients harboring the fusion. However, the molecular pathways have been elucidated for only a few fusions, in part because of the labor-intensive nature of the required functional assays. Therefore, we developed a domain-based network approach to infer the pathways of a fusion. Molecular interactions of a fusion are first predicted by using its protein domain composition, and its associated pathways are then inferred from these molecular interactions. We demonstrated the capabilities of this approach by primarily applying it to the well-studied BCR-ABL1 fusion. The approach was also applied to two undruggable fusions in sarcoma, EWS-FL1 and FUS-DDIT3. We successfully identified known genes and pathways associated with these fusions and satisfactorily validated these predictions using several benchmark sets. The predictions of EWS-FL1 and FUS-DDIT3 also correlate with results of high-throughput drug screening. To our best knowledge, this is the first approach for inferring pathways of fusions.

## Author summary

We present a computational framework, FusionPathway, to infer the oncogenesis pathways of a fusion and help develop therapeutic strategies in these pathways for patients harboring the fusion. In this work, we successfully validated the capabilities of this approach through its application to the well-studied BCR-ABL1 fusion and two undruggable fusions in sarcoma, EWS-FL1 and FUS-DDIT3. Especially, the predictions of EWS-FL1 and FUS-DDIT3 correlate well with results of high-throughput drug screening in sarcoma cells. Therefore, FusionPathway can be an effective method to infer pathways and potential therapeutic targets that are associated with those undruggable fusions. Our results of BCR-ABL1 also suggest that FusionPathway may be able to help elucidate pathway-dependent mechanisms of resistances to those kinase fusion-targeting therapies and develop strategies to overcome the resistances. In addition, the developed R package of FusionPathways (https://github.com/perwu/FusionPathway/) can help people easily apply our approach to study other important fusions in cancer.

This is a *PLOS Computational Biology* Methods paper.

## Introduction

Gene fusions resulting from chromosome rearrangements and chimeric RNAs generated by trans-splicing or read-through events play important roles in cancer. Recently, the advent of massively parallel sequencing has accelerated the rate of the discovery of fusions across multiple cancer types [1]. Fusions, especially druggable kinase fusions, may serve as therapeutic targets in cancer [2]. However, many identified fusions are not druggable, such as EWS-FLI1 [3] and C11orf95-RELA [4]. In addition, drug resistance to fusion-targeted therapies, such as resistance to imatinib in chronic myeloid leukemia (CML), may develop in some patients owing to mutations that render a drug unable to bind to its targeted fusion or to the activation of compensatory pathways [5]. A better understanding of the molecular pathways of fusions would facilitate the development of therapeutic strategies for patients who harbor these fusions. However, owing to the labor-intensive nature of the validation assays required, few studies have investigated the pathways of novel fusions [6]. Therefore, computational methods for predicting the pathways of fusions would greatly help understand their functions in oncogenesis and develop therapeutic strategies.

Numerous methods to annotate the functional impact of point mutations by mutation rate, reading frame, and evolutionarily conserved regions have been developed [7]. In particular, some methods incorporate pathway-level information and gene expression data to assess the functional impact of mutated genes [8]. These pathway-based methods suggest that important mutated genes deregulate the expression levels of their interacting partners and pathways, whereas passenger mutations have little impact. However, these approaches may not be applicable to fusions, whose interacting partners and pathways underlying their oncogenesis remain obscure. Several computational approaches that can prioritize fusion drivers in sequencing data have been proposed recently. Wu *et al*. [9] developed a fusion centrality metric to score fusions under the assumption that a fusion is more likely to be an oncogenic driver if its parental genes act like hubs in a molecular network. Two other approaches, Oncofuse [10] and Pegasus [11], use supervised learning methods to identify fusion drivers by a feature space composed of protein domains, reading frame annotations, and protein-protein interaction partners. However, these approaches cannot elucidate the pathways of a fusion.

Several computational approaches for predicting molecular interactions of proteins on the basis of domain information have been developed [12]. A fusion retains some of the functional domains of its parental genes [13]. Thus, we reason that molecular interactions of a fusion can be inferred given the composition of its protein domains. Latysheva *et al*. [14] recently unified all the protein interactions of the parental proteins of a fusion and found a gene fusion may rewire the protein interaction network in cancer through connecting proteins that did not previously interact in the network. But, they did not consider that a fusion may not inherit some protein interactions from its parental proteins because of losing some of their protein domains. In addition, a very recent study [15] presented a method, “ChiPPI”, to identify preserved protein-protein interactions of fusions using the domain-domain co-occurrence scores. Yet, this method does not help uncover the pathways of a specific fusion. Herein, we propose a domain-based network approach to infer the molecular interactions and pathways associated with a fusion and explore potential therapeutic targets in these pathways. We demonstrated the capabilities of our approach by applying it to the BCR-ABL1 fusion and two undruggable fusions, EWS-FL1 and FUS-DDIT3.

## Methods

### Domain-based network approach

**Fig 1:**
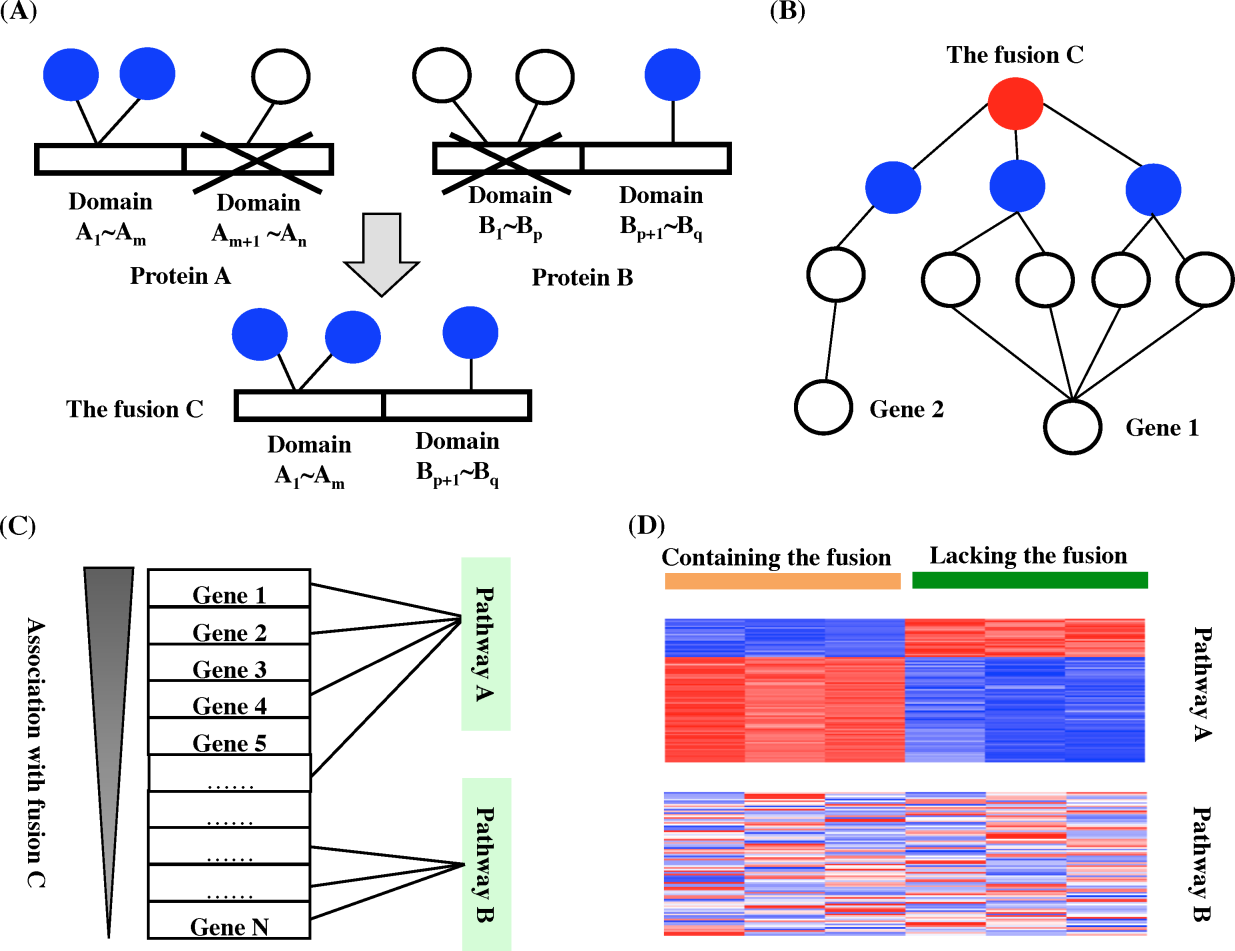
The domain-based network approach. (A) Prediction of molecular interactions of fusion C, whose parental proteins are proteins A and B that contain n and q domains respectively, denoted A_1_~A_n_ and B_1_~B_q_. Fusion C comprises domains A_1_~A_m_ and B_q+1_~B_q_. Blue and white nodes represent proteins that have protein-protein interactions or genes that have protein-DNA interactions with proteins A or B. Only the proteins or genes represented by the blue nodes can interact with fusion C because the domains of fusion C can bind to their protein domains (for protein-protein interactions) or DNA sequence close to their promoters (for protein-DNA interactions). (B) Calculation of the functional association score of each gene with fusion C (red node) through predicted molecular interaction partners (blue nodes). Genes that are more functionally associated with these predicted partners (e.g., gene 1) are also more functionally associated with fusion C than other genes (e.g., gene 2) are. (C) Prediction of pathways associated with fusion C. Genes are ranked on the basis of their association scores with fusion C. Pathway A, which enriches genes with higher association scores, is more functionally associated with fusion C than is pathway B. (D) Gene expression data from samples containing or lacking a fusion are leveraged to identify pathways deregulated by the fusion.

Our proposed domain-based network approach is illustrated in Fig 1. First, a fusion protein consists of the domains of its parental proteins, therefore we hypothesized that the fusion protein retains some of the molecular interactions of its parental proteins. Given the fusion’s domain composition, we can predict the protein-protein or protein-DNA interactions of the fusion (Fig 1A). Protein-protein interactions result primarily from the binding of a modular domain of one protein to the domains of another protein [16]. The protein-protein interaction partners of the fusion’s parental proteins, whose domains interact with the domains of the fusion, are predicted to be the protein-protein interaction partners of the fusion. In addition, some fusions act as transcription factors to deregulate gene transcriptions through protein-DNA interactions. Because protein-DNA interactions mainly occur when a transcription factor composed of DNA-binding domains binds to a motif within promoters of its target genes to regulate their expression, we simply assumed that a fusion containing DNA-binding domains of its parental genes would be able to deregulate the transcriptional regulation interactions of its parental genes. A set of protein-protein interactions, a set of protein domain-domain interactions, and a set of transcriptional regulation interactions were used to predict molecular interactions of fusions (S1 Text).

Second, we reasoned that genes that are close to these predicted molecular interactions in the network would be functionally associated with the fusion, a principle known as “guilt by association” [17] (Fig 1B). Our approach uses the random walk with restart [18] to calculates the functional relatedness of each gene with the fusion (termed fusion-association score) on the basis of the relative location of each gene to the predicted molecular interaction partners of the fusion in a compiled gene network (S1 Text). On the basis that the hypothetical random walkers diffuse along the links of the network from the predicted molecular interaction partners, genes that are close to the predicted molecular interaction partners will be those most often visited by the random walkers during their random walks. Given a network with n nodes (i.e., genes in the gene network), the random walk with restart is defined as:

$$p_{t+1}=(1‑\gamma )Tp_{t}+\gamma p_{0}$$ (1)

where *p*_*0*_ is the initial probability vector in which equal probabilities are assigned to the starting nodes (i.e., the predicted molecular interaction partners), *p*_*t*_ is the probability vector containing the probabilities of the nodes at step *t*, *γ* is the restarting probability, and *T* is the transition matrix, which is a column-normalized adjacency matrix of the network. Starting from the set of nodes in the network, the walker will iteratively move from the current nodes to randomly selected neighbor nodes or return to the starting nodes. When iteratively reaching stability (i.e., when the change between *p*_*t*_ and *p*_*t+1*_ is below 10e-30), the probability vector can present the association scores of all genes to the starting genes. Thus, genes with higher association scores are also more associated with the fusion.

Third, we considered pathways that enrich genes with high fusion-association scores are also associated with the fusion (Fig 1C). We ranked all the genes on the basis of their fusion-association scores. Gene set enrichment analysis (GSEA) [19–20], which can evaluate the genes of a pathway for their distribution in the ordered gene list, was then used to identify pathways functionally associated with a fusion (termed GSEA association analysis). However, because the gene network used in the prediction contains molecular interactions across different cellular statuses, not all predicted associated pathways are deregulated by a fusion in a specific cellular condition. By leveraging gene expression data from samples that have or lack a fusion, we can identify pathways that are deregulated by the fusion (Fig 1D). The deregulation level of an associated pathway can be also determined using GSEA (termed GSEA deregulation analysis). The truncated product method [21] was used to combine the p values of each pathway generated from the GSEA association and GSEA deregulation analyses to identify pathways that are both highly associated with the fusion and significantly deregulated by it in a specific cellular condition. In the truncated product method, the product score *W* of the two p values (*p*_*i*_) that do not exceed a fixed *τ* value (*τ* was set to 0.01 for both p values) can be calculated as:

$$W=\prod_{i=1}^{2}p_{i}^{I(p_{i\leq \tau })}$$ (2)

where *I*(.) is the indicator function. The probability of *W* for *w*<1 can be evaluated by conditioning on the number, *k*, of the *p*_*i*_’s less than *τ*:

Pr(W≤w)=∑k=12Pr(2k)(1‑τ)2‑k(w∑s=0k‑1(klnτ‑lnw)ss!I(w≤τk)+τkI(w>τk)) (3)

### Gene sets for prediction evaluation

Literature co-citation data have been widely used to infer gene-gene and gene-disease functional associations [22–23]. Therefore, we compiled two types of benchmark gene sets from literature citation data to comprehensively evaluate each fusion prediction. First, we collected 378 genes cited in at least two papers related to BCR-ABL1, 29 genes cited in at least two papers related to EWS-FLI1, and 11 genes cited in at least one papers related to FUS-DDIT3 (As only few literature citations of FUS-DDIT3 were found, a lower criteria was applied here) to evaluate the predictions of genes functionally associated with these fusions. We downloaded all PubMed identification numbers of articles related to each fusion. These numbers were then cross-referenced with the gene citation information from Entrez Gene (ftp://ftp.ncbi.nih.gov/gene/), which is composed of genes and corresponding cited literature. Second, using the similar method, we collected 416 genes cited in at least two papers related to CML, 98 genes cited in at least two papers related to Ewing’s sarcoma, and 61 genes cited in at least one paper related to myxoid liposarcoma to evaluate whether fusion-associated genes in our prediction play a role in the oncogenesis of the specific cancer type. We also used a set of 328 cancer-related genes compiled from KEGG cancer pathways to evaluate the association between cancer pathways and the three fusions.

### Drug targets for prediction evaluation

To evaluate our predictions of therapeutic targets in fusion-associated pathways, we collected 68 target genes of drugs that have been tested in clinical trials or used for the treatment of CML and 67 drug target genes for Ewing sarcoma (S1 Table and S3 Table). These target genes were manually collected from the literature and available public databases (detailed in S2 Text and S3 Text). Furthermore, we also evaluated our predictions using target genes of sensitive compounds identified in high-throughput screening for two Ewing’s sarcoma cell lines (TC32 and TC71) and three myxoid liposarcoma cell lines (MLS-1765-92, MLS-402-91, and MLS-DL221). The material and method of high-throughput screening are detailed in the S1 Text. We identified 76 sensitive compounds for Ewing cell lines and 48 sensitive compounds for myxoid liposarcoma cell lines. With the incorporation of known drug target data, we respectively found 60 of the 76 sensitive compounds for Ewing cells and 38 of the 48 sensitive drugs for the myxoid liposarcoma cells have known target genes. Totally, we have 197 targets of sensitive compounds for Ewing cells and 161 targets for myxoid liposarcoma cells (S4 Table and S5 Table).

## Results

### Prediction of BCR-ABL1-associated pathways

A variety of breakpoints in BCR and ABL1 rearrangements generate BCR-ABL1 chimeric proteins with different domain compositions, of which the three major variants (p185, p210, and p230) occur in different types of leukemia [24]. These variants may be associated with tissue-specific spatial organization of genomes [25]. The ABL1 portion of all three variants contains tandem SRC homology, the tyrosine kinase domains, SH3 binding sites, a DNA-binding domain, and an actin-binding domain [24]. However, the BCR portions of the three variants differ. p230 contains the largest amount of BCR sequence, including a calcium-binding domain, a Gap-Rac domain, a coiled-coil oligomerization domain, a serine/threonine kinase domain, a Dbl homology domain, and a pleckstrin homology domain. p210 and p185 both lose the Gap-Rac domains, but p185 also loses the Dbl homology and pleckstrin homology domains. We first applied our approach to the p210 BCR-ABL1, which is the most commonly associated with CML.

**Fig 2:**
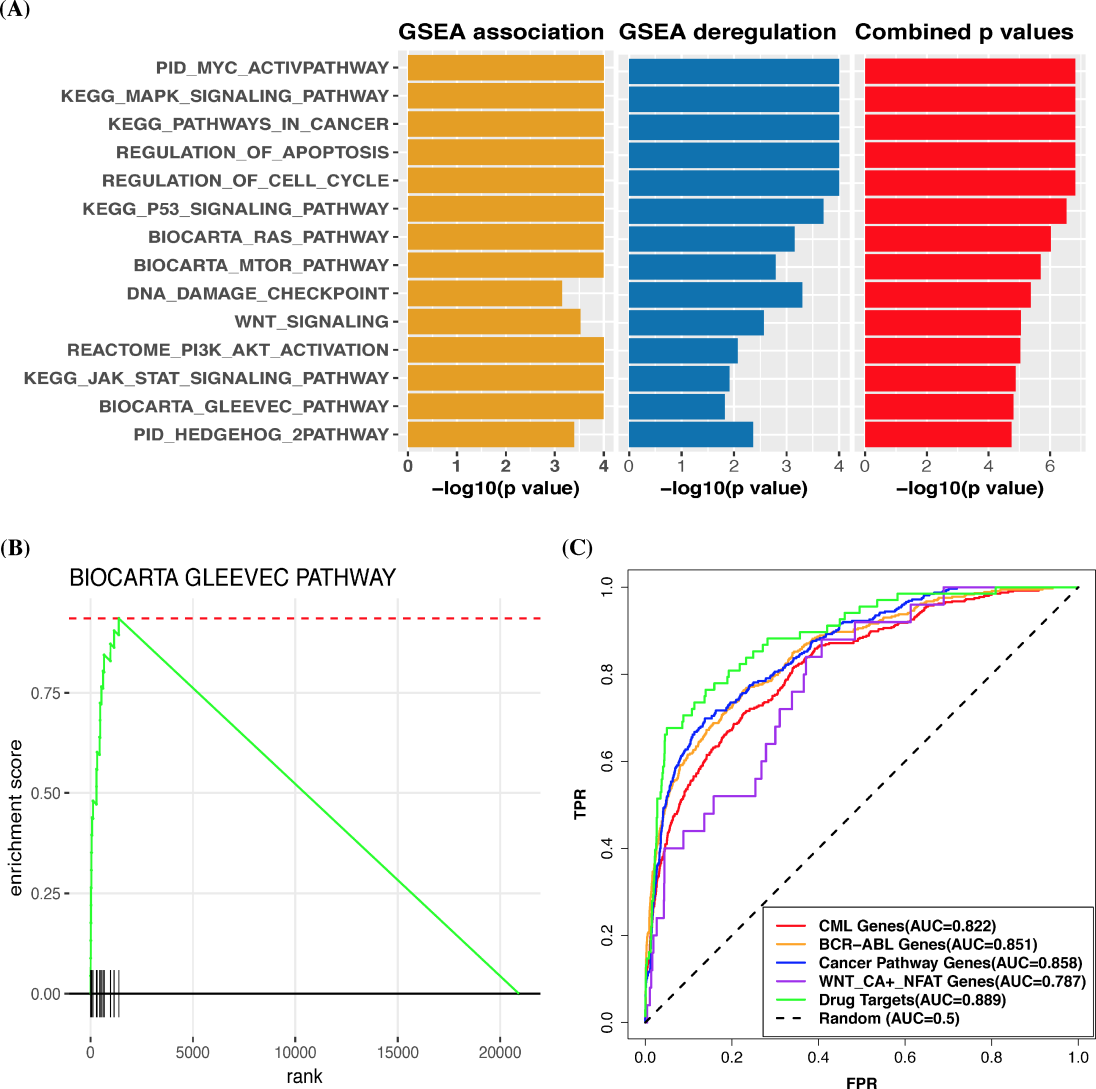
Evaluation of p210 BCR-ABL1 prediction using known BCR-ABL1 pathways and benchmark gene sets. (A) Bar plot of some statistically significant pathways that are associated with BCR-ABL1 in our prediction. A bar in the graph represents the statistically significance of a given pathway in GSEA association analysis, GSEA deregulation analysis, or combination analysis using the truncated product method. The statistically significances are presented in the graph as–log10(p-value). (B) GSEA association plot of the Gleevec pathway in our prediction. GSEA evaluates the genes of the pathway for their distribution in the ordered gene list generated by our association prediction. (C) ROC curves for five benchmark gene sets: 26 Wnt/Ca+/NFAT pathway genes (denoted WNT_CA+_NFAT genes), 1150 genes cited with BCR-ABL1 in the literature (denoted BCR-ABL genes), 1240 genes cited with CML in the literature (denoted CML genes), 328 genes categorized in the KEGG cancer pathways (denoted cancer pathway genes), and 68 target genes of compounds that have been tested in clinical trials or used for the treatment of imatinib-resistant CML (denoted drug targets). Genes in these five gene sets were treated as positive instances, and the remaining genes were treated as negative instances. TPR indicates true positive rate; FPR, false positive rate; and AUC, area under the ROC curve.

Our approach first predicted the protein-protein interaction partners of p210 BCR-ABL1. Many proteins that are known to physically interact with BCR-ABL1, such as GRB2, CRKL [26], and SOCS1/3 [27], were in our prediction (S2 Text). This supports our hypothesis that a fusion retains some molecular interactions of its parental genes. Next, we used these predicted protein interaction partners to infer BCR-ABL1-associated pathways. GSEA association analysis revealed that several known BCR-ABL1 pathways are highly associated with BCR-ABL1 in our prediction (Fig 2A), such as imatinib pathway (Fig 2B shows its GSEA association plot), JAK-STAT signaling pathway [26], DNA damage pathway [28], Hedgehog pathway [29], and p53 pathway [30]. We also analyzed the gene expression data of three CML cell lines and their imatinib-treated cells [31] and examined the deregulation of these pathways using GSEA. The combination analysis of the GSEA association and GSEA deregulation analyses shows that these pathways are both highly associated with BCR-ABL1 and significantly deregulated upon its inhibition (Fig 2A). Our approach also predicted most of 26 Wnt/Ca+/NFAT pathway genes identified by RNAi-based screen with imatinib in CML cells [32] are associated with BCR-ABL1 (Fig 2C). Specifically, CAMK2B, one of the top 5% of genes associated with BCR-ABL1 in our prediction, is a target of cyclosporin A, which can sensitize BCR-ABL-positive leukemia to BCR-ABL inhibitors [32]. This implies that our approach can help identify therapeutic targets associated with a fusion (detailed in the following section).

**Fig 3:**
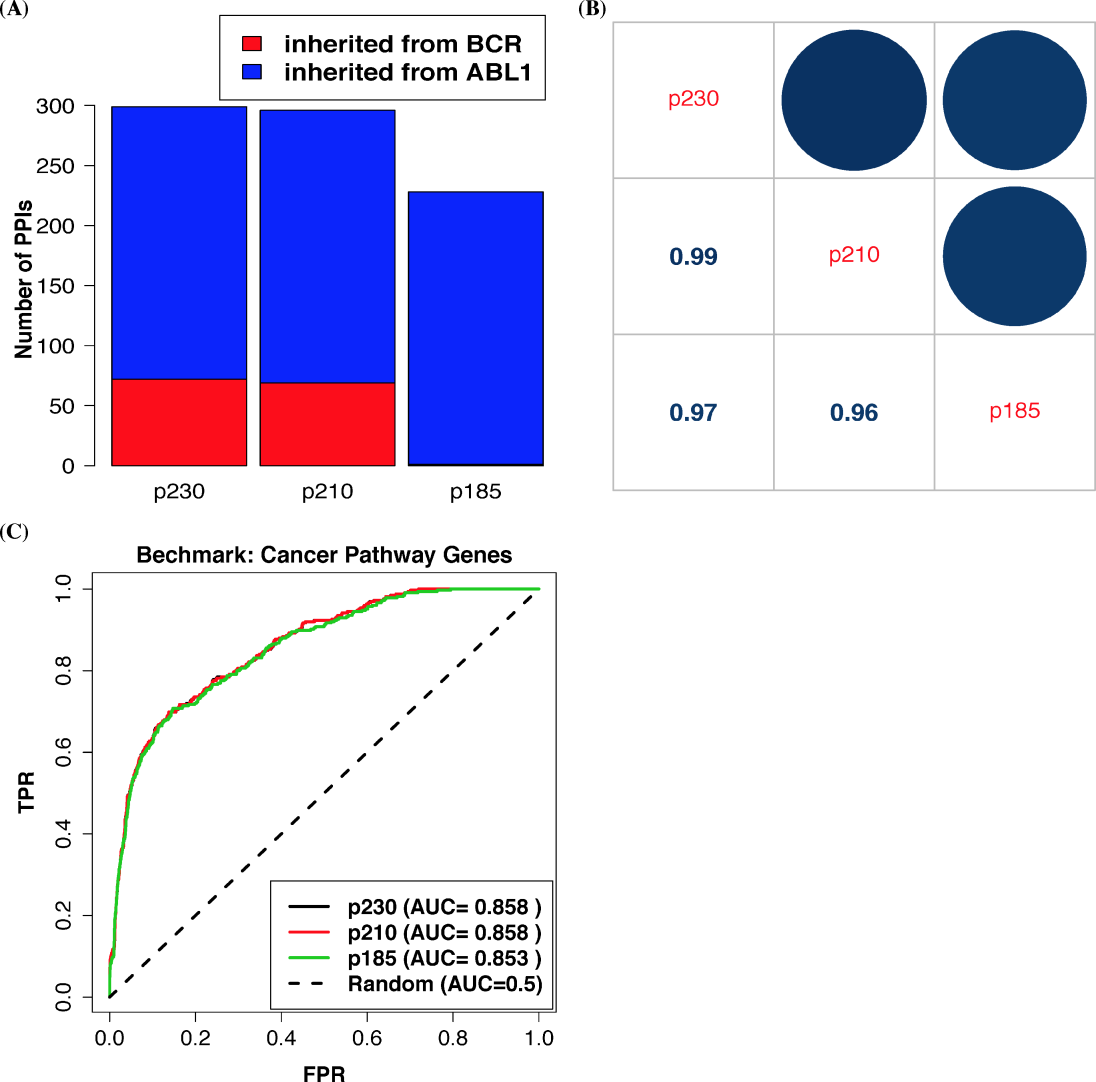
Prediction evaluation of three known BCR-ABL1 variants (p185, p210, and p230). (A) Number of predicted protein-protein interaction partners of the three BCR-ABL1 variants. (B) Correlations of pathway prediction of the three BCR-ABL1 variants. GSEA association scores of categorized pathways in the MSigDB database (category C2) were used for calculating spearman correlations. (C) Oncogenic impact evaluation of the three BCR-ABL1 variants using genes categorized in the KEGG cancer pathway.

In addition, we also collected several literature-based benchmark gene sets to comprehensively evaluate our prediction, including BCR-ABL1 related genes, CML related genes, and cancer pathway genes (Fig 2C). These evaluations using the ROC analysis (Fig 2C) and the other two statistical tests (S2 Text) all showed that most of these benchmark genes were highly associated with BCR-ABL1 in our prediction. Furthermore, our predictions of BCR-ABL1 were also correlated with several data-driven gene signatures associated with BCR-ABL1 and CML (S1 Fig). These results all indicate that our approach can successfully predict cancer-related genes and pathways that are associated with BCR-ABL1. Furthermore, we also compared our predictions of the three BCR-ABL1 variants. We found that the three variants had similar numbers of predicted protein interactions (Fig 3A) and the three variants had almost the similar pathway predictions (Fig 3B). Prediction evaluation using the 328 cancer pathway genes (Fig 3C) also indicated that these variants would have almost the same oncogenic impact. Similar results were observed in evaluations using different benchmark sets (S2 Fig). Some studies also have shown that these three variants are equally potent in inducing a CML-like disease in transplanted mice [33].

### Potential therapeutic targets in BCR-ABL1–associated pathways

Inhibition of BCR-ABL1 fusion by imatinib has proven to be a very successful treatment for CML with the BCR-ABL fusion. However, some patients develop imatinib resistance owing to the emergence of BCR-ABL1 point mutations. Several second-generation drugs that target both BCR-ABL1 and its associated pathways thus have been developed for the treatment of imatinib-resistant CML [5]. Therefore, we also used p210 BCR-ABL1 as an example to illustrate the capability of our approach to identify therapeutic targets associated with a fusion.

In our literature review, we collected 68 target genes of 24 drugs that have been in clinical trials or used for the treatment of CML (S1 Table). We successfully predicted that most of these 68 genes are highly associated with BCR-ABL1 (Fig 2C). This indicates that our approach could help develop therapeutic strategies that target the pathways associated with a fusion. Therefore, we also mapped the drug target data from the DGIdb database [34] to the top 10% of genes associated with BCR-ABL1 in our prediction. We identified some of these compounds, such as ruxolitinib, regorafenib, and arsenic trioxide, have been shown to have an anti-cancer effect in CML cells or other cancer cells and could be potential treatments for CML patients (Table 1). Ruxolitinib, a JAK1/2 inhibitor, is used for the treatment of intermediate or high-risk myelofibrosis. Studies *in vitro and in vivo* support the use of ruxolitinib for the treatment of CML [35–36]. Regorafenib, a multikinase inhibitor, has been approved for the treatment of advanced gastrointestinal stromal tumors and metastatic colorectal cancer [37]. Regorafenib can target several BCR-ABL1–associated genes, such as KIT. Some studies showed that KIT signaling governs differential sensitivity to tyrosine kinase inhibitors in mature and primitive CML progenitors [38]. Arsenic trioxide has been approved for the treatment of acute promyelocytic leukemia [39], and recent work suggests that arsenic trioxide is also a treatment option for CML [40].

**Table 1:** Selected compounds whose targets are in the top 5% of genes functionally associated with *BCR-ABL1*.

Compound	Target genes (top 5%)	Citation
Ruxolitinib	*JAK2*, *JAK1*	[35–36]
Regorafenib	*KIT*, *NTRK1*, *ABL1*, *RAF1*, *PDGFRB*, *KDR*, *FRK*, *RET*, *EPHA2*, *BRAF*, *FGFR1*, *PDGFRA*, *MAPK11*, *TEK*, *FLT1*, *FGFR2*, *FLT4*	[37]
Arsenic trioxide	*JUN*, *MAPK3*, *AKT1*, *MAPK1*, *IKBKB*, *CCND1*	[39–40]
Sorafenib	*KIT*, *RAF1*, *PDGFRB*, *KDR*, *RET*, *BRAF*, *FGFR1*, *FLT1*, *FLT3*, *FLT4*	[41–42]
Pazopanib	*KIT*, *PDGFRB*, *ITK*, *KDR*, *SH2B3*, *PDGFRA*, *FLT1*, *FLT4*	[43]
Celecoxib	*PDPK1*	[44–45]
Geldanamycin	*HSP90AA1*, *HSP90AB1*	[46]
Vorinostat	*HDAC1*, *HDAC2*, *HDAC3*	[47]

We also integrated the gene expression data of three CML cell lines and their imatinib-treated CML cells [31] with the top genes associated with BCR-ABL1 in our prediction to prioritize targets whose inhibition can have synergistic effects with imatinib. About 500 genes were significantly up-regulated in imatinib-treated CML cells (fold change >1.5 and p < 0.05), and 50 of them were in the top 10% of genes in our prediction (S2 Table). Some of the 50 genes are potential therapeutic targets. For example, inhibition of JAK2 can overcome imatinib drug resistance in CML [35]. Inhibition of CSNK2A2 by CX-4945 also exhibits anti-tumor activity in chronic lymphocytic leukemia [48]. BCL6, which has been shown to be up-regulated in response to treatment with imatinib, represents a novel defense mechanism enabling leukemia cells to survive despite imatinib treatment [31]. In addition, studies also showed that CBL-B is required for the leukemogenesis mediated by BCR-ABL through the negative regulation of bone marrow homing [49] and that CML with the CBL-B mutation is resistant to imatinib [50]. Although some of these predicted targets in our analysis are currently undruggable, developing RNAi-based therapies would enable us to target these genes [51].

### Prediction of two sarcoma-associated fusions

Sarcomas have greater than 50 histological subtypes, which arise in bone, cartilage or connective tissues. Currently, standard chemotherapy, radiation, and surgery are the only available treatments for most of sarcomas. Thus, alternative therapeutic strategies are urgently needed to improve survival rate and quality of life for sarcoma patients. Several types of sarcomas are driven by the presence of specific fusion mutations, such as SYT-SSX in synovial sarcoma, EWS-FLI1 in Ewing’s sarcoma, FUS-DDIT3 in myxoid liposarcoma [3], and EWS-WT1 in Desmoplastic Small Round Cell Tumor (DSRCT) [52]. These fusions can produce tumor-specific proteins; thus are potential targets for the development of specific therapies for these sarcomas. However, most of these fusions are transcriptional regulators, making them more challenging as drug targets [2]. Understanding the pathways that are associated with these fusions would reveal alternative therapeutic strategies. In this work, we also applied our approach to two undruggable sarcoma fusions, EWS-FLI1 and FUS-DDIT3.

**Fig 4:**
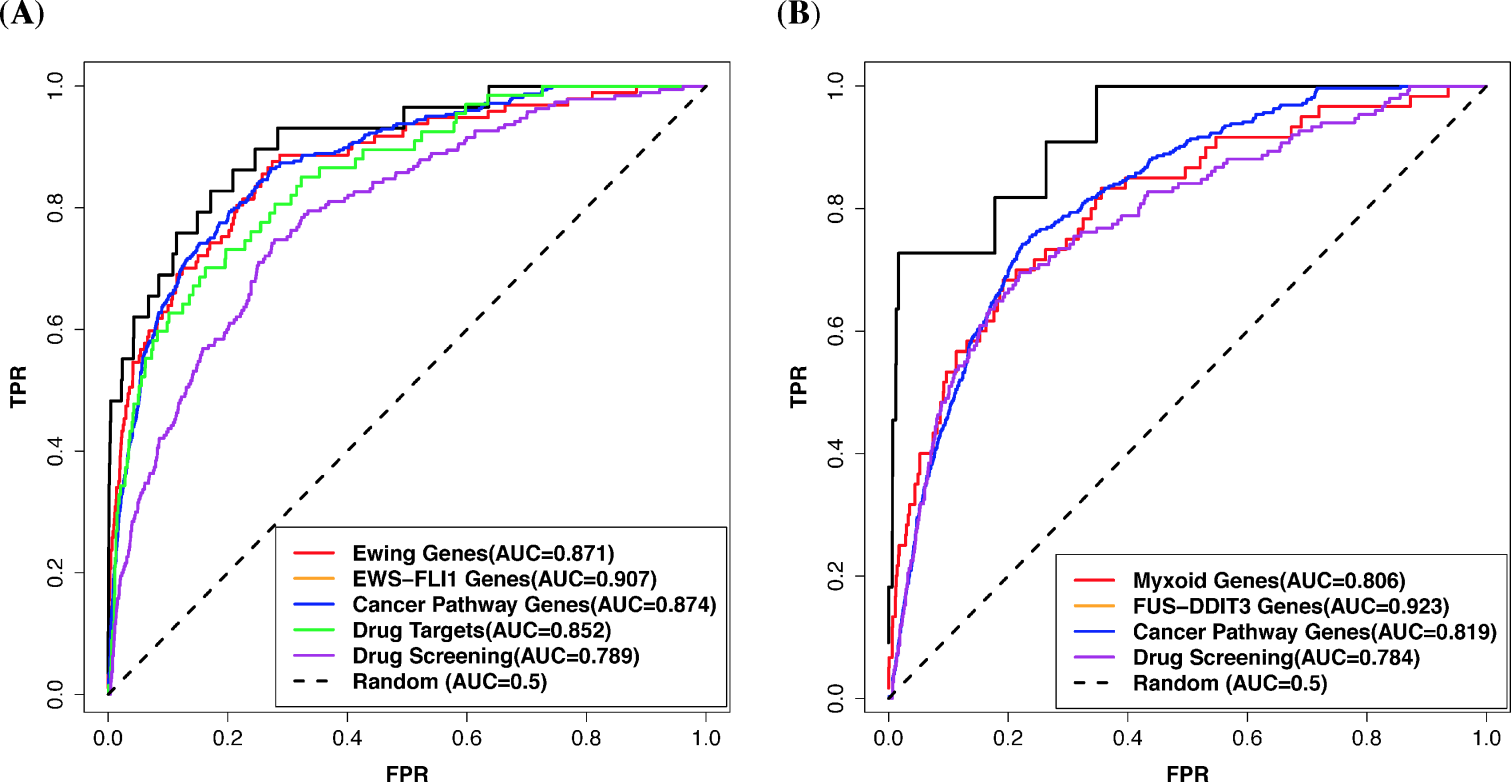
Prediction evaluation of two undruggable fusions in sarcoma using benchmark gene sets. (A) EWS-FLI1 fusion. (B) FUS-DDIT3 fusion. Target gene sets of identified sensitive compounds of Ewing sarcoma and Myxoid liposarcoma in high-throughput screening assay are denoted drug screening. Other benchmark gene sets have similar denotations as Fig 2.

We successfully predicted the pathways that are known to be functionally associated with these two fusions, for instance, WNT [53], IGF1 signaling [54], and PDGFR pathways [55] for EWS-FLI1 (S3 Fig), and adipocytokine signaling [56], DNA damage [57], NF-kB pathways [58], and FGFR pathway [59] for FUS-DDIT3 (S5 Fig). Several pathways that were recently found to be associated with EWS-FLI1 are also identified by our approach, such as chromatin remodeling [60], splicing pathways [61], and CRM1-dependent nuclear export pathway [62] (S3 Fig). Our predictions were also satisfactorily validated by several literature-based benchmark sets (Fig 4, S3 Text, and S4 Text). In addition, several data-driven gene signatures associated with Ewing’s sarcoma and EWS-FLI1 were also correlated well with our predictions (S4 Fig). These results indicate that our approach can successfully identify most of known genes and pathways associated with the two fusions.

High-throughput screening has been widely used in drug-discovery processes to rapidly identify compounds that are active againsts of particular pathways in tumours [63]. Therefore, we also compared our predictions of two fusions respectively with the high-throughput screening results in two Ewing’s sarcoma and three myxoid liposarcoma cell lines. The known target genes of the identified sensitive compounds in the screening were used to evaluate our predictions (S4 Table and S5 Table). The ROC analysis of the evaluation shows that our predictions correlate well with the screening results (Fig 4). We also found that 51 of the 60 (85%) sensitive compounds for Ewing cells have at least one target gene ranked among the top 10% of those predicted to be associated with EWS-FLI1, and 37 of the 38 (97.37%) sensitive compounds for the myxoid liposarcoma cells have at least one target gene ranked among the top 10% genes associated with FUS-DDIT3. Specifically, some studies showed that Ewing sarcoma cells are sensitive to PARP1 inhibition [64–65], and our high-throughput screening results also showed that the two Ewing cells are sensitive to a PARP1 inhibitor, BMN 673. Our prediction also successfully identify PARP1 is one of top 5% genes that are functionally associated with EWS-FLI1 in the prediction. In addition, we also collected target genes of drugs that have been tested in clinical trials or used for the treatment of Ewing sarcoma to evaluate our prediction of EWS-FLI1. The evaluation shows that most of these target genes are functionally associated with EWS-FLI1 in our prediction (Fig 4A). Furthermore, Myxiod liposarcoma has been shown to be high sensitive to a novel chemotherapeutic agent, Trabectedin [66]. The recent data suggested Trabectedin may block the transactivation of FUS-DDIT3 [67]. We also found that our prediction of FUS-DDIT3 was correlated with several data-driven signatures associated with Trabectedin (S6 Fig). These results show that our approach can be used to identify therapeutic targets in the associated pathways of these two undruggable fusions and can be a way of in-silico drug screening. Other results of these two fusions are detailed in the S3 Text and S4 Text.

### Overview of the R package “FusionPathway”

**Fig 5:**
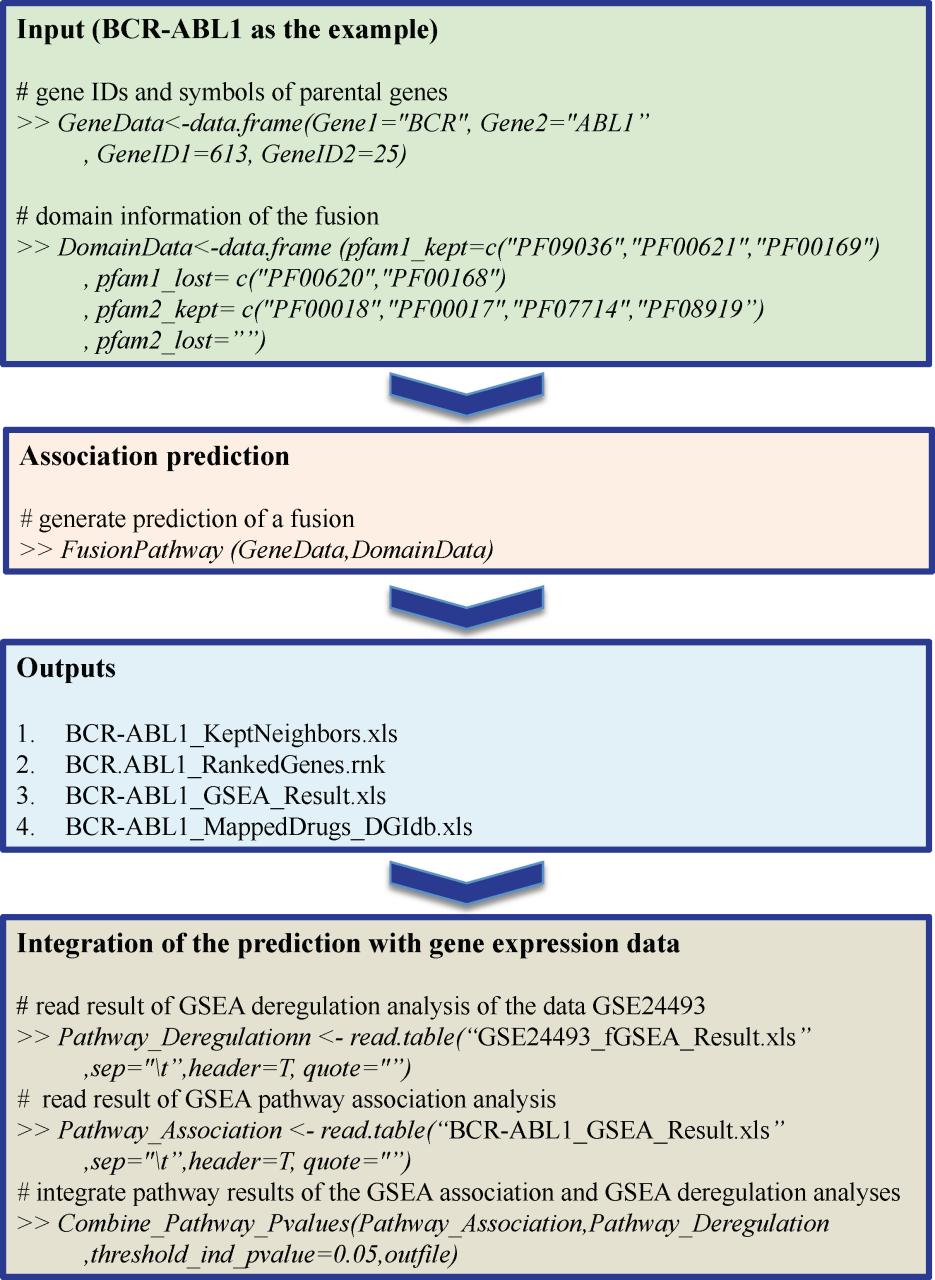
Workflow for the FusionPathway package.

The R package “FusionPathway” is available in the GitHub repository (https://github.com/perwu/FusionPathway/) and runs in R environment. Installation of the R package is described in the GitHub page of “FusionPathway”. The scripts and data for generating the results of three examples demonstrated in this manuscript are also provided in the package. The general workflow of the package is shown in Fig 5, in which we took BCR-ABL1 as an example. First, the basic input data includes two data frame objects: “GeneData” and “DomainData”. “GeneData” should contain Entrez gene IDs and gene symbols of the two parental genes whereas “DomainData” should contain Pfam protein domain IDs of the two parental genes, which are retained and lost in a fusion. Second, the F*usionPathway* function in the package was used to generate the association prediction of a fusion. Third, the outputs include four files: 1. a list of predicted the molecular interaction partners of a fusion; 2. an ordered gene list ranked by the fusion-association prediction; 3. results of GSEA pathway association analysis; 4. results of mapped drugs. Those top genes associated with a fusion can be selected using the values of rank percentage (e.g. 10%), the top pathways associated with a fusion can be chosen using the p values of the GSEA association analysis, and potential drugs can be prioritized using the selected top genes. Fourth, by integrating gene expression data from samples that have or lack a fusion with our association prediction, we can identify pathways that are both highly associated with a fusion and significantly deregulated by it in a specific cellular condition. The fGSEA R package [20] (also included in the package) can be first used to do GSEA pathway deregulation analyses based on the gene list ordered by p-values of the differential gene expression analysis. The *Combine_Pathway_Pvalues* function can then be used to integrate pathway results of the GSEA association and GSEA deregulation analyses. We will make continual improvements to the package for its performance and usability.

## Discussion

### Prediction of fusion molecular interactions

In our domain-based network approach, we hypothesized that a fusion retains some of the functional domains and its cognate molecular interactions from its parental genes. Indeed, we found that most known BCR-ABL1 protein-protein interactions were inherited from BCR and ABL1. Thus, the molecular functions of a fusion would be mainly associated with the functions of its parental genes. This implies that a fusion plays an important role in oncogenesis if at least one of its parental genes is associated with cancer. Some also believe that, rather than demolishing the entire machinery and creating a new version to trigger oncogenesis, cancer cells normally make modifications to existing cellular mechanisms [68]. Several recent studies support this view. Two studies [9,14] found that the parental genes of many known cancer fusions are also cancer-related and act as hubs in molecular networks. Other studies also revealed that important fusions in cancer normally retain functional domains associated with oncogenesis from their parental genes [13]. However, a fusion may have novel functions and molecular interactions that are absent in its parental genes because of the shuffling of different active sites and binding domains or the generation of new domain combinations [69]. Several computational approaches can predict the novel molecular interactions of proteins on the basis of multiple domain combinations [70]. These approaches can be integrated into our model to predict the novel protein interactions of a fusion based on its novel domain combinations.

### Prediction of potential therapeutic targets and drugs

Our results reveal that our approach can help identify potential therapeutic targets in the pathways associated with a fusion. However, not all of the top genes associated with a fusion in our prediction can be therapeutic targets because their associations with the fusion may relate to different types of phenotypic effects [71]. Therefore, integrating other high-throughput data, such as gene expression data and RNAi screening, with the predicted top genes can help prioritize potential therapeutic targets. For instance, by integrating gene expression data, we successfully identified genes whose inhibition can sensitize imatinib-resistant CML cells to treatment. In addition, RNAi screening has been used to identify critical genes that control cancer-related phenotypes without using any prior biological information [72]. However, when no prior biological information is used, RNAi screening must be used to assess all genes. In contrast, integration of top genes in our prediction with RNAi screening can greatly reduce the search space.

With the incorporation of drug target data, our approach also can help identify compounds that may have action in pathways associated with a fusion. However, some of these identified compounds may be ineffective in the treatment of patients harboring the fusion [73]. First, the drug-binding affinities are target-dependent. In addition, the mechanisms of action of some drugs are unclear. Finally, most drugs act against multiple targets, and inhibition of these targets may lead to different types of phenotypic effects, both positive and negative [71]. Therefore, evaluating the possible therapeutic effects of the identified drugs is difficult. However, our approach could serve as a quick way to initially screen a small number of potential drugs subjected to further evaluation, such as high-throughput screening. The good correlations between our predictions of two sarcoma fusions and the high-throughput screening results support this view. This indicates that integration of our approach predictions can help reduce the search space in high-throughput screenings [74]. Moreover, if the targets of some drugs that have been already approved by the U.S. Food and Drug Administration are highly ranked in the prediction, such drugs could be repositioned for the treatment of cancers harboring specific fusions.

Diverse drug resistance mechanisms, including pathway-dependent mechanisms (e.g., target reactivation through secondary mutation, downstream activation, or bypass activation) and pathway-independent mechanisms (e.g., tumor microenvironment perturbation), remain major problems for targeted therapies [75]. Combinations of drugs targeting the associated pathways may prevent drug resistance [76]. Our results of BCR-ABL1 suggest that our approach may help elucidate pathway-dependent mechanisms of resistances to those kinase fusion-targeting therapies and develop strategies to overcome the resistances.

### Comparison to the other methods

Very recently, a method, ChiPPI, was proposed to identify preserved protein-protein interactions of fusion proteins [15]. We found there are two major differences between our study and ChiPPI. First, ChiPPI only predicts protein-protein interactions of fusions while our method can predict both of protein-protein interactions and protein-DNA interactions of fusions. Thus, our method could more accurately uncover molecular mechanisms of those fusions that act as transcription factors to deregulate pathways mainly through protein-DNA interactions. Second, rather than doing pathway analysis of each fusion, ChiPPI performed a pathway enrichment analysis of all the predicted interactors of all the fusions in the three major disease types, leukemia/lymphoma, sarcoma or solid tumors, to identify the over-presented pathways in each of the three cancer types. In contrast, our method used the guilt-by-association method to identify pathways that are associated (either directly and undirectly interact) with an individual fusion. Our method can help understand molecular mechanisms of a specific fusion and explore therapeutic targets in these pathways for patients harbouring the fusion.

Both our method and CHiPPI predicted which protein interactions of parental proteins will be kept in a fusion given the domain compositions of a fusion. But, our method used a set of curated protein domain-domain interactions (S1 Text) to predict protein interactions of fusions while ChiPPI predicts interactions based on domain-domain co-occurrence scores that were calculated *de novo* based on all collected interactions in the BioGrid database [77]. In addition, our method predicted protein interactions of fusions from a set of protein-protein interactions compiled from several expert-curated databases (S1 Text) while ChiPPI made predictions based on protein interactions from BioGrid. We here used 33 known protein interactors of BCR-ABL1 (S2 Text) that were compiled from literature to compare BCR-ABL1 (p210) predictions of our method and ChiPPI. We found that our method predicted 30 of the 33 known protein interactions while ChiPPI predicted 24 the known interactions. However, we need to emphasize that it is difficult to evaluate prediction performance between our method and ChiPPI because it requires robust positive and negative interactions of a fusion, which does not currently exist.

### Summary

Our approach is not without limitations. Because it is based on incomplete molecular interaction data, our approach cannot be used to determine the biological functions of fusions whose parental genes or functional domains have not yet been well characterized. In addition, our approach is not able to predict novel molecular interactions that are absent in its parental genes because of the shuffling of different active sites and binding domains or the generation of new domain combinations or other mechanisms. However, our approach will improve over time as more data are generated. Regardless, our results indicate that our approach can be an effective method for inferring the pathways of a fusion and identifying potential therapeutic targets in these pathways. This will greatly help develop therapeutic strategies for patients who harbor undruggable fusions and for those whose disease is resistant to fusion-targeted therapies.

## Supporting information

S1 Text Supporting information for network data and drug screens. (DOCX)

S2 Text Supporting information for the *BCR-ABL1* prediction. (DOCX)

S3 Text Supporting information for the *EWS-FLI1* prediction. (DOCX)

S4 Text Supporting information for the *FUS-DDIT3* prediction. (DOCX)

S1 Fig Prediction evaluation Of *BCR-ABL1* using data-driven gene signatures. The three data-driven gene signatures were collected from several previous works (detailed in S2 Text).
(TIFF)

S2 Fig Prediction evaluation of *BCR-ABL1* variants using the four benchmark gene sets. The three known *BCR-ABL1* variants are denoted “p185”, “p210”, and “p230” respectively. (a) genes co-cited with *BCR-ABL1* in literature. (b) CML associated genes. (c) target genes of drugs that have been already in clinical trials or used for treatment of CML (d) 26 *Wnt/Ca+/NFAT* pathway genes, which were identified by RNAi-based synthetic lethal screen with imatinib mesylate in CML cells.
(TIFF)

S3 Fig Bar plot of some statistically significant pathways that are associated with *EWS-FLI1* in our prediction. A bar in the graph represents the statistically significance of a given pathway in GSEA association analysis, GSEA deregulation analysis, or combination analysis using the truncated product method. The statistically significances are presented in the graph as–log_10_(p-value).
(TIFF)

S4 Fig Prediction evaluation of *EWS-FLI1* using data-driven gene signatures that are associated with *EWS-FLI1* or Ewing’s sarcoma. The four data-driven gene signatures were collected from several previous works (detailed in S3 Text).
(TIFF)

S5 Fig Bar plot of some statistically significant pathways that are associated with *FUS-DDIT3* in our prediction. A bar in the graph represents the statistically significance of a given pathway in GSEA association analysis, GSEA deregulation analysis, or combination analysis using the truncated product method. The statistically significances are presented in the graph as–log_10_(p-value).
(TIFF)

S6 Fig Evaluation of *FUS-DDIT3* prediction using data-driven gene signatures that are associated with Trabectedin. The four data-driven gene signatures associated with Trabectedin were collected from several previous works (detailed in S4 Text).
(TIFF)

S1 Table Compounds that have been in clinical trials or used for treatment of CML. We complied 68 target genes of 24 compounds that have been already in clinical trials and used for treatment of CML from several review papers and drug databases (detailed in S2 Text).
(DOCX)

S2 Table Top 10% *BCR-ABL1*- associated genes that are significantly up-regulated upon imatinib treatment. (DOCX)

S3 Table Compounds that have been in clinical trials and used for treatment of Ewing’s sarcoma. We complied 67 target genes of 22 drugs that have been already in clinical trials and used for treatment of Ewing’s sarcoma from several review papers and drug databases (detailed in S3 Text).
(DOCX)

S4 Table Sensitive compounds for Ewing’s sarcoma cell lines and their target genes. 76 sensitive compounds were identified in two Ewing’s sarcoma cell lines (TC32 and TC71) using the high-throughput screening assay. Target genes of these sensitive compounds were complied from available public databases, and 60 of the 76 compounds have known target genes. Totally, we have 197 drug targets of the 60 compounds.
(DOCX)

S5 Table Sensitive compounds for myxoid liposarcoma cell lines and their target genes. 48 sensitive compounds were identified in three Myxoid liposarcoma cell lines (MLS-1765-92, MLS-402-91, and MLS-DL221) using the high-throughput screening assay. Target genes of these sensitive compounds were complied from available public databases, and 38 of the 48 compounds have known target genes. Totally, we have 161 drug targets of the 38 compounds.
(DOCX)
