# Supplementary material for: FusionPathway: Prediction of pathways and therapeutic targets associated with gene fusions in cancer
Source: PLoS Comput Biol. 2018 Jul 24;14(7):e1006266. doi: 10.1371/journal.pcbi.1006266 (PMC6075785; doi:10.1371/journal.pcbi.1006266)
Supplement: S1 Text — (DOCX) [file pcbi.1006266.s001.docx]

**Supporting Information For Network Data And Drug Screens**

**NETWORK DATA USED FOR PREDICTIONS**

A set of protein-protein interactions and a set of protein domain-domain interactions were used for predicting protein interactions of fusions. The protein-protein interaction set was compiled from several expert-curated databases, including the Human Protein Reference Database (Mathivanan et al., 2008), INstruct (Meyer et al., 2013), and Reactome (Vastrik et al., 2007). The protein domain-domain interactions set was also compiled from several databases, including the DOMAIN (Yellaboina et al., 2011), IDDI (Kim et al., 2012), and INstruct (Meyer et al., 2013). A total of 29,5441 protein-protein interactions involving 17,313 genes and 29,216 domain-domain interactions involving 14,936 genes were used in the analysis. In addition, a set of transcriptional regulation interactions and a set of DNA-binding domains were used for predicting transcriptional regulation interactions of fusions. The transcriptional regulation interaction set was compiled from several expert-curated and highly reliable databases, including the Transcriptional Regulatory Element Database (Zhao et al., 2005), ITFP (Zheng et al., 2008), HTRIdb database (Bovolenta et al., 2012), and TRRUST (Han et al., 2015). The set of DNA-binding domains was downloaded from the DBD database (Kummerfeld and Teichmann, 2006). A total of 130,672 transcriptional regulation interactions involving 18,982 genes were used in the analysis. Also, a gene network that was compiled from several curated and predicted biological networks (Zhao et al., 2005; Jiang et al., 2007; Vastrik et al., 2007; Mostafavi et al., 2008; Zheng et al., 2008; Cerami et al., 2011; Bovolenta et al., 2012; Meyer et al., 2013; Han et al., 2015) was used in network guilt-by-association method to calculate the functional association score of each gene with a fusion. This network is more suitable than the aforementioned protein interaction and transcriptional regulation interaction networks for calculating gene functional association because it contains not only direct physical interactions but also broader gene-gene functional relationships within pathways.

**DRUG SCREEN**

High-throughput screening assay was applied to evaluate inhibition activities of more than 1000 compounds in two Ewing’s sarcoma cell lines (TC32 and TC71), three myxoid liposarcoma cell lines (MLS-1765-92, MLS-402-91, and MLS-DL221), and other sarcoma cell lines that are not used in this study. Briefly, growth curves of cells were first generated by seeding cells in two-fold dilutions (2000 to 125 cells/well) in 50 uL of growth media per 6-8 technical replicate wells. Cell counts were obtained daily by counting nuclei using an automated confocal microscope, GE INCell 6000 Analyzer (GE Healthcare Life Sciences, Pittsburgh, PA). Subsequently, cellular growth rate was calculated and an appropriate plating density was selected in order for cells to be in log phase growth during treatment and for cells to be 70-80% confluent or less at 72 h after compound treatment. Then, 400 – 3000 cells per well, as determined by growth assay were plated in 50uL of growth media per well and kept overnight at 37ºC and 5% CO2 prior to the addition of 50 nL compound by pin transfer from 1000x DMSO stocks using a Beckman Coulter Biomek FX (Beckman Coulter, Brea, CA) equipped with a 50 nL pin toll from V&P Scientific (V&P Scientific, Inc., San Diego, CA). Compounds from the Custom Clinical (147 compounds), NCI Oncology (112 compounds) and SelleckBioactive collections (1150 compounds) were screened. After washing the cells, DAPI-stained nuclei were segmented and counted using the InCell Developer (GE Healthcare Life Sciences). Staurosporine or etoposide treatments were run as internal positive controls on all screening plates. A z-prime statistic (Zhang et al., 1999) was calculated for each assay plate using the on-plate drug-treated positive and DMSO treated negative control wells. A minimum significant ratio (MSR) (Eastwood et al., 2006) was calculated for the eight-point control concentration response curves and compared from all of the assay plates on the screen. Z-prime statistics for each screening data were evaluated to remove those that were too low. A comparison of the well-to-well standard deviation over the two replicate screens was also performed to evaluate the screens for high variability between runs. The numerical results from image analysis of the microtiter plates are first normalized to intra-plate controls. Any plates that failed validation were repeated. Dose-response characteristics are determined by fitting a 4-parameter logistic (“Hill”) equation to the fraction affected (fa) metric of compound response values, and the area under the curve (AUC) is computed using numerical integration. AUC values are then normalized to a range of 0 to 1 (AUCn) by dividing the AUC values by the number of logs over which the compounds were tested (5 logs, from 10-10 to 10-5). Compounds were divided into four broad classes, depending upon the values of AUCn and the mean-squared error associated with the curve fit, as follows: Class 1 drugs were those with an AUCn of at least 0.7 and an MSE of less than 0.01. Class 2 drugs failed Class 1 conditions but had an AUCn of at least 0.4 and an MSE of less than 0.025. Class 3 drugs failed both Class 1 and 2 conditions but had an AUCn of at least 0.1, an MSE of less than 0.05 and a difference of at least 0.3 between max_fit_fa and min_fit_fa. Class 4 drugs were those that failed to pass the conditions for Classes 1 through 3.

Compounds that were categorized as Class 1 in at least two cell lines of a tumor type were considered to be sensitive compounds for the tumor type. We identified 76 sensitive compounds for both of two Ewing cell lines and 48 sensitive drugs in at least two of the three MLS cell lines. Target genes of these sensitive drugs were complied from several available public databases, including PharmGKB (Hodge et al., 2007), the Therapeutic Target Database (Zhu et al., 2010), and DrugBank (Knox et al., 2011), and DGIdb (Griffith et al., 2013). We found 60 of the 76 sensitive compounds for Ewing cell lines have known target genes, and 38 of the 48 sensitive drugs for the MLS cell lines. Totally, we have 197 and 161 drug targets of these compounds respectively for Ewing’s sarcoma and myxoid liposarcoma. These targets covered a wide range of processes implicated in oncogenesis of Ewing’s sarcoma and myxoid liposarcoma, and were used to evaluate our predictions. These target genes are listed in S4 Table and S5 Table respectively in this additional file.

**REFERENCES**

Bovolenta LA, Acencio ML, Lemke N. HTRIdb: an open-access database for experimentally verified human transcriptional regulation interactions. BMC Genomics. 2012;13:405.

Cerami EG, Gross BE, Demir E, Rodchenkov I, Babur O, Anwar N, et al. Pathway Commons, a web resource for biological pathway data. Nucleic Acids Res. 2011;39(Database issue):D685-90.

Eastwood BJ, Farmen MW, Iversen PW, Craft TJ, Smallwood JK, Garbison KE, et al. The minimum significant ratio: a statistical parameter to characterize the reproducibility of potency estimates from concentration-response assays and estimation by replicate-experiment studies. J Biomol Screen. 2006;11(3): 253-61.

Griffith M, Griffith OL, Coffman AC, Weible JV, McMichael JF, Spies NC, et al. DGIdb: mining the druggable genome. Nat Methods 2013;10(12):1209-10.

Han H, Shim H, Shin D, Shim JE, Ko Y, Shin J. TRRUST: a reference database of human transcriptional regulatory interactions. Sci Rep. 2015;5:11432.

Hodge AE, Altman RB, Klein TE. The PharmGKB: integration, aggregation, and annotation of pharmacogenomic data and knowledge. Clin Pharmacol Ther. 2007;81(1):21-4.

Jiang C, Xuan Z, Zhao F, Zhang MQ TRED: a transcriptional regulatory element database, new entries and other development. Nucleic Acids Res. 2007;35(Database issue):D137-40.

Kim Y, Min B, Yi GS. IDDI: integrated domain-domain interaction and protein interaction analysis system. Proteome Sci. 2012;10 Suppl 1:S9.

Knox C, Law V, Jewison T, Liu P, Ly S, Frolkis A, et al. DrugBank 3.0: a comprehensive resource for 'omics' research on drugs. Nucleic Acids Res 2011;39(Database issue):D1035-41.

Kummerfeld SK, Teichmann SA. DBD: a transcription factor prediction database. Nucleic Acids Res. 2006;34(Database issue):D74-81.

Mathivanan S, Ahmed M, Ahn NG, Alexandre H, Amanchy R, Andrews PC, et al. Human Proteinpedia enables sharing of human protein data. Nat Biotechnol. 2008;26(2):164-7.

Meyer MJ, Das J, Wang X, Yu H. INstruct: a database of high-quality 3D structurally resolved protein interactome networks. Bioinformatics. 2013;29(12):1577-9.

Mostafavi S, Ray D, Warde-Farley D, Grouios C, Morris Q. GeneMANIA: a real-time multiple association network integration algorithm for predicting gene function. Genome Biol 2008;9:s4.

Vastrik I, D'Eustachio P, Schmidt E, Gopinath G, Croft D, de Bono B, et al. Reactome: a knowledge base of biologic pathways and processes. Genome Biol. 2007;8:R39.

Yellaboina S, Tasneem A, Zaykin DV, Raghavachari B, Jothi R. DOMINE: a comprehensive collection of known and predicted domain-domain interactions. Nucleic Acids Res. 2011;39(Database issue):D730-5.

Zhao F, Xuan Z, Liu L, Zhang MQ TRED: a Transcriptional Regulatory Element Database and a platform for in silico gene regulation studies. Nucleic Acids Res. 2005;33(Database issue):D103-7.

Zhang JH, Chung TD, Oldenburg KR. A simple statistical parameter for use in evaluation and validation of high throughput screening assays. J Biomol Screen.1999;4(2):67-73.

Zheng G, Tu K, Yang Q, Xiong Y, Wei C, Xie L, et al. ITFP: an integrated platform of mammalian transcription factors. Bioinformatics. 2008;24(20):2416-7.

Zhu F, Han B, Kumar P, Liu X, Ma X, Wei X, et al. (2010) Update of TTD: Therapeutic target database. Nucleic Acids Res 38(Database issue): D787-791.
