# Supplementary material for: FusionPathway: Prediction of pathways and therapeutic targets associated with gene fusions in cancer
Source: PLoS Comput Biol. 2018 Jul 24;14(7):e1006266. doi: 10.1371/journal.pcbi.1006266 (PMC6075785; doi:10.1371/journal.pcbi.1006266)
Supplement: S2 Text — (DOCX) [file pcbi.1006266.s002.docx]

**Supporting Information For The BCR-ABL1 Prediction**

**KNOWN PROTEIN-PROTEIN INTERACTIONS OF *BCR-ABL1* IN OUR PREDICTION**

| **Gene** | **Reference** |
| --- | --- |
| *ABI1* | Titz et al., 2010; Zhuang et al., 2011 |
| *BLK* | Zhang et al., 2012 |
| *CBL* | Brehme et al., 2009; Kolch and Pitt, 2010 |
| *CRK* | Jankowski et al., 2012 |
| *CRKL* | Kolch and Pitt, 2010; Jankowski et al., 2012 |
| *DOK1* | Bhat et al., 1998; Titz et al., 2010 |
| *DOK2* | Titz et al., 2010 |
| *DOK3* | Lemay et al., 2000 |
| *FES* | Lionberger and Smithgall, 2000 |
| *GAB2* | Cilloni and Saglio, 2012 |
| *GRB2* | Kolch and Pitt, 2010; Cilloni and Saglio, 2012 |
| *GRB10* | Bai et al., 1998; Okino et al., 2005 |
| *HCK* | Warmuth et al., 1997; Stanglmaier et al., 2003 |
| *INPPL1* | Brehme et al., 2009 |
| *IRS1* | Traina et al., 2003 |
| *JAK2* | Xie et al., 2001 |
| *LRRK1* | Titz et al., 2010 |
| *NCK1* | Titz et al., 2010 |
| *NEDD9* | Titz et al., 2010 |
| *PIK3R1* | Ren et al., 2005; Brehme et al., 2009; Kolch and Pitt, 2010 |
| *PIK3R2* | Brehme et al., 2009 |
| *PTPN6* | Liedtke et al., 1998 |
| *PTPN11* | Kolch and Pitt, 2010; Rubbi et al., 2011 |
| *RASA1* | Qiu et al., 2012 |
| *RHOA* | Sahay et al., 2008 |
| *SHC1* | Kolch and Pitt, 2010 |
| *SOCS1* | Qiu et al., 2012 |
| *SOCS3* | Qiu et al., 2012 |
| *SOS1* | Fan and Goff, 2000; Sini et al., 2004 |
| *SOS2* | Fan and Goff, 2000 |
| *SRC* | Danhauser-Riedl et al., 1996; Rubbi et al., 2011 |
| *UBASH3B* | Kolch and Pitt, 2010; Brehme et al., 2009 |

**PREDICTION EVALUATION USING LITERATURE-BASED BENCHMARK GENE SETS AND** **DATA-DRIVEN GENE SIGNATURES, WHICH ARE ASSOCIATED WITH *BCR-ABL1* OR CML**

We collected several literature-based benchmark gene sets to comprehensively evaluate our prediction using the ROC analysis, such as *BCR-ABL1* related genes (Fig 2C in the main paper). We also applied two other methods evaluate our predictions: gene set enrichment analysis (GSEA) and the Mann-Whitney-Wilcoxon test. GSEA can evaluate a set of genes for their distribution in the ordered gene list against random permutations. The AUC of a ROC curve is directly connected to the Mann-Whitney U-Statistic (Mason and Graham, 2012). Therefore, we also used the Mann-Whitney-Wilcoxon test to determine the significance of all the ROC curves. The following table lists p-values of all evaluations using the two methods (please note that the minimal p.value of fGSEA output is 0.0001. So, pval =0.0001 indicates the real p.value<=0.0001).

| **Benchmarks** | **Wilcoxon.pval** | **GSEA.pval** |
| --- | --- | --- |
| CML_Genes | 2.6925e-109 | 0.0001 |
| BCR_ABL1_Genes | 1.004e-119 | 0.0001 |
| WNT-CA-NFAT_Genes | 6.8204e-07 | 0.0014 |
| CancerPathway_Genes | 4.1257e-109 | 0.0001 |
| Drug_TargetGenes | 1.323e-28 | 0.0001 |

In addition, three data-driven gene signatures that are associated with *BCR-ABL1* or chronic myeloid leukemia (CML) were also used to evaluate our prediction of p210 *BCR-ABL1*. These genes sets include 45 genes that are significantly differentially expressed in pre-B lymphoblastic leukemia cells with the *BCR-ABL1* fusion, compared to normal pre-B lymphocytes (Klein et al., 2006), 1,498 genes that are significantly differentially expressed in CD34+ cells isolated from bone marrow of CML patients, compared to those from normal donors (Diaz-Blanco et al., 2007), and 34 genes that are significantly differentially expressed in HL-60 cells (acute myeloid leukemia, AML) by expression of p210 *BCR-ABL* fusion (Ray et al., 2004). The evaluations indicate that our prediction correlates well with these gene signatures (S1 Fig).

**TARGET GENES OF COMPOUNDS THAT HAVE BEEN ALREADY IN CLINICAL TRIALS AND USED FOR TREATMENT OF CML**

To evaluate our prediction of therapeutic targets in *BCR-ABL1* associated pathways, we manually collected 68 target genes of 24 compounds that have been already in clinical trials and used for treatment of CML (S1 Table). These data were compiled from 3 highly accessed review papers (Walz and Sattler, 2006; Weisberg et al., 2007; Woessner et al., 2011), Cancer Drug Information in National Cancer Institute (<http://www.cancer.gov/cancertopics/druginfo/leukemia#dal4>), and public drug database: PharmGKB (Hodge et al., 2007), the Therapeutic Target Database (Zhu et al., 2010), and DrugBank (Knox et al., 2011), and DGIdb (Griffith et al., 2013). The result of the ROC curve evaluation is shown in Fig 2C of the main article. The high AUC value of the ROC curve indicates that our approach can successfully predict potential therapeutic targets in associated pathways of *BCR-ABLI1*.


**REFERENCES**

Bai RY, Jahn T, Schrem S, Munzert G, Weidner KM, Wang JY, Duyster J. The SH2-containing adapter protein GRB10 interacts with BCR-ABL. Oncogene.

Bhat A, Johnson KJ, Oda T, Corbin AS, Druker BJ. Interactions of p62(dok) with p210(bcr-abl) and Bcr-Abl-associated proteins. J Biol Chem. 1998;273(48):32360-8.

Brehme M, Hantschel O, Colinge J, Kaupe I, Planyavsky M, Köcher T, et al. Charting the molecular network of the drug target Bcr-Abl. Proc Natl Acad Sci U S A. 2009;106(18):7414-9.

Cilloni D, Saglio G. Molecular pathways: BCR-ABL. Clin Cancer Res. 2012;18(4): 930-7.

Danhauser-Riedl S, Warmuth M, Druker BJ, Emmerich B, Hallek M. Activation of Src Kinases p53/56 lyn and p59 hck by p210 bcr/abl in Myeloid Cells. Cancer Res. 1996;56:3589-96.

Diaz-Blanco E, Bruns I, Neumann F, Fischer JC, Graef T, Rosskopf M, et al. Molecular signature of CD34(+) hematopoietic stem and progenitor cells of patients with CML in chronic phase. Leukemia. 2007;21(3):494-504.

Fan PD, Goff SP. Abl interactor 1 binds to sos and inhibits epidermal growth factor- and v-Abl-induced activation of extracellular signal-regulated kinases. Mol Cell Biol. 2000;20(20):7591-601.

Griffith M, Griffith OL, Coffman AC, Weible JV, McMichael JF, Spies NC, et al. DGIdb: mining the druggable genome. Nat Methods 2013;10(12):1209-10.

Hodge AE, Altman RB, Klein TE. The PharmGKB: integration, aggregation, and annotation of pharmacogenomic data and knowledge. Clin Pharmacol Ther. 2007;81(1):21-4.

Jankowski W, Saleh T, Pai MT, Sriram G, Birge RB, Kalodimos CG. Domain organization differences explain Bcr-Abl's preference for CrkL over CrkII. Nat Chem Biol. 2012;8(6):590-6.

Klein F, Feldhahn N, Herzog S, Sprangers M, Mooster JL, Jumaa H, et al. BCR-ABL1 induces aberrant splicing of IKAROS and lineage infidelity in pre-B lymphoblastic leukemia cells. Oncogene. 2006;25(7):1118-24.

Knox C, Law V, Jewison T, Liu P, Ly S, Frolkis A, et al. DrugBank 3.0: a comprehensive resource for 'omics' research on drugs. Nucleic Acids Res 2011;39(Database issue):D1035-41.

Lemay S, Davidson D, Latour S, Veillette A. Dok-3, a novel adapter molecule involved in the negative regulation of immunoreceptor signaling. Mol Cell Biol. 2000;20(8):2743-54.

Liedtke M, Pandey P, Kumar S, Kharbanda S, Kufe D. Regulation of Bcr-Abl-induced SAP kinase activity and transformation by the SHPTP1 protein tyrosine phosphatase. Oncogene. 1998;17(15):1889-92.

Lionberger JM, Smithgall TE. The c-Fes protein-tyrosine kinase suppresses cytokine-independent outgrowth of myeloid leukemia cells induced by Bcr-Abl. Cancer Res. 2000;60(4):1097-103.

Mason SJ and Graham NE. Areas beneath the relative operating characteristics (ROC) and relative operating levels (ROL) curves: Statistical significance and interpretation. Journal of the Royal Meteorological Society. 2002;128(584):2145-66.

Okino K, Konishi H, Doi D, Yoneyama K, Ota Y, Jin E, et al. Up-regulation of growth factor receptor-bound protein 10 in cervical squamous cell carcinoma. Oncol Rep. 2005;13(6):1069-74.

Qiu X, Guo G, Chen K, Kashiwada M, Druker BJ, Rothman PB, Chen JL. A requirement for SOCS-1 and SOCS-3 phosphorylation in Bcr-Abl-induced tumorigenesis. Neoplasia. 2012;14(6):547-58.

Ray S, Lu Y, Kaufmann SH, Gustafson WC, Karp JE, Boldogh I, et al. Genomic mechanisms of p210BCR-ABL signaling: induction of heat shock protein 70 through the GATA response element confers resistance to paclitaxel-induced apoptosis. J Biol Chem. 2004;279(34):35604-15.

Ren R. Mechanisms of BCR–ABL in the pathogenesis of chronic myelogenous leukaemia. Nat Rev Cancer. 2005;5(3):172-83.

Rubbi L, Titz B, Brown L, Galvan E, Komisopoulou E, Chen SS, Low T, et al. Global phosphoproteomics reveals crosstalk between Bcr-Abl and negative feedback mechanisms controlling Src signaling. Sci Signal. 2011;4(166):ra18.

Sahay S, Pannucci NL, Mahon GM, Rodriguez PL, Megjugorac NJ, Kostenko EV, Ozer HL, Whitehead IP. The RhoGEF domain of p210 Bcr-Abl activates RhoA and is required for transformation. Oncogene 2008;27(14):2064-71.

Sini P, Cannas A, Koleske AJ, Di Fiore PP, Scita G. Abl-dependent tyrosine phosphorylation of Sos-1 mediates growth-factor-induced Rac activation. Nat Cell Biol. 2004;6(3):268-74.

Stanglmaier M, Warmuth M, Kleinlein I, Reis S, Hallek M. The interaction of the Bcr-Abl tyrosine kinase with the Src kinase Hck is mediated by multiple binding domains. Leukemia. 2003;17(2):283-9.

Titz B, Low T, Komisopoulou E, Chen SS, Rubbi L, Graeber TG. The proximal signaling network of the BCR-ABL1 oncogene shows a modular organization, Oncogene 2010;29(44):5895-910.

Traina F, Carvalheira JB, Saad MJ, Costa FF, Saad ST. BCR-ABL binds to IRS-1 and IRS-1 phosphorylation is inhibited by imatinib in K562 cells. FEBS Lett. 2003;535(1-3):17-22.

Walz C, Sattler M. Novel targeted therapies to overcome imatinib mesylate resistance in chronic myeloid leukemia (CML). Crit Rev Oncol Hematol 2006;57:145-64.

Warmuth M, Bergmann M, Priess A, Häuslmann K, Emmerich B, Hallek M. The Src family kinase Hck interacts with Bcr-Abl by a kinase-independent mechanism and phosphorylates the Grb2-binding site of Bcr. J Biol Chem. 1997;272(52):33260-70.

Weisberg E, Manley PW, Cowan-Jacob SW, Hochhaus A, Griffin JD. Second generation inhibitors of BCR-ABL for the treatment of imatinib-resistant chronic myeloid leukaemia. 2007;Nat Rev Cancer 7(5):345-56.

Willems SM, Schrage YM, Bruijn IH, Szuhai K, Hogendoorn PC, Bovée JV. Kinome profiling of myxoid liposarcoma reveals NF-kappaB-pathway kinase activity and casein kinase II inhibition as a potential treatment option. Mol Cancer. 2010;9:257.

Woessner DW, Lim CS, Deininger MW. Development of an effective therapy for chronic myelogenous leukemia. Cancer J. 2011;17(6):477-86.

Xie S, Wang Y, Liu J, Sun T, Wilson MB, Smithgall TE, Arlinghaus RB. Involvement of Jak2 tyrosine phosphorylation in Bcr-Abl transformation. Oncogene. 2001;20(43):6188-95.

Zhang H, Peng C, Hu Y, Li H, Sheng Z, Chen Y, et al. The Blk pathway functions as a tumor suppressor in chronic myeloid leukemia stem cells. Nat Genet. 2012;44(8):861-71.

Zhu F, Han B, Kumar P, Liu X, Ma X, Wei X, et al. (2010) Update of TTD: Therapeutic target database. Nucleic Acids Res 38(Database issue): D787-791.

Zhuang C, Tang H, Dissanaike S, Cobos E, Tao Y, Dai Z. CDK1-mediated phosphorylation of Abi1 attenuates Bcr-Abl-induced F-actin assembly and tyrosine phosphorylation of WAVE complex during mitosis. J Biol Chem. 2011;286(44):38614-26.
