# Supplementary material for: FusionPathway: Prediction of pathways and therapeutic targets associated with gene fusions in cancer
Source: PLoS Comput Biol. 2018 Jul 24;14(7):e1006266. doi: 10.1371/journal.pcbi.1006266 (PMC6075785; doi:10.1371/journal.pcbi.1006266)
Supplement: S3 Text — (DOCX) [file pcbi.1006266.s003.docx]

**Supporting Information For The EWS-FLI1 Prediction**

**INTRODUCTION**

Ewing sarcoma is a highly malignant bone and soft tissue tumor that occurs most frequently in children and adolescents. The 5-year overall survival rate for patients with localized Ewing sarcoma is approximately 70%, but the rate drops to less than 10% for those with metastasis (Martín Liberal et al., 2012). Currently, standard chemotherapy, radiation, and surgery are the only available treatments for Ewing sarcoma (Subbiah and Anderson, 2011). New therapeutic strategies based on the biology of Ewing sarcoma are needed to improve outcomes. In 85% of cases, Ewing sarcoma is associated with a unique gene rearrangement between the *EWS* gene and an *FLI1* transcription factor gene (Arvand and Denny, 2001). The *EWS-FLI1* fusion protein, which is composed of the transcriptional activator *EWS* domain and the DNA-binding domain of *FLI1*, acts as a transcription factor whose expression contributes to cell transformation and tumor progression (Janknecht, 2005). Many studies have revealed that *EWS-FLI1* promotes tumor development and progression by inducing its downstream genes, such as *ID2*, *CCND1*, *PDGFC*, *IGF1*, *MYC*, *CCND-1*, and *NKX2-2*, which are involved in pathways related to cell proliferation and survival (Ordóñez et al., 2009; Kovar, 2010; Jully and Rajkumar, 2012; Kelleher and Thomas, 2012). Therapeutics strategies targeting the pathways associated with *EWS-FLI1* may thus have clinical value.

**TARGET GENES OF COMPOUNDS THAT HAVE BEEN ALREADY IN CLINICAL TRIALS AND USED FOR TREATMENT OF EWING’S SARCOMA**

To evaluate our prediction of therapeutic targets in *EWS-FLI1* associated pathways, we manually collected 67 target genes of 22 drugs that have been already in clinical trials and used for treatment of Ewing’s sarcoma (S3 Table). These data were compiled from several literature (DuBois et al., 2010; Subbiah and Anderson, 2011; Anderson et al., 2012; Chandhanayingyong et al., 2012; Jully and Rajkumar, 2012; Kelleher and Thomas, 2012; Lissat et al., 2012; Martín Liberal et al., 2012) and public drug database: PharmGKB (Hodge et al., 2007), the Therapeutic Target Database (Zhu et al., 2010), DrugBank (Knox et al., 2011), and DGIdb (Griffith et al., 2013). These drugs mainly target on known *EWS-FLI1* associated pathways, including *IGF1* signaling, *PI3K/AKT/MTOR* pathway, *P53* pathway, and histone deacetylase. The result of the ROC curve evaluation is shown in Fig 4A of the main text. The high AUC value of the ROC curve indicates that our approach can successfully predicts therapeutic targets in associated pathways of *EWS-FLI1*.


**PREDICTION OF KNOWN *EWS-FLI1* PATHWAYS**

We used our domain-based network approach to predict pathways that are functionally associated with *EWS-FLI1*. GSEA association analysis revealed that several known *EWS-FLI1* pathways are highly functionally associated with *EWS-FLI1* in our prediction (S3 Fig), such as *Wnt* (Hu-Lieskovan et al., 2005; Navarro et al., 2010), *IGF1* signaling (Prieur et al., 2004), *PDGFR* (Uren et al., 2003), *MAPK* (Benini et al., 2004; Chandhanayingyong et al., 2012), *PI3K/AKT* (Benini et al., 2004), *p53* pathways (Lissat et al., 2012). Gene expression data of Ewing's sarcoma cell lines upon *EWS-FLI1* knockdown (Bilke et al., 2013) were also used to determine deregulation of these pathways. GSEA deregulation analyses show these pathways are also significantly deregulated upon *EWS-FLI1* knockdown (S3 Fig). In addition, several pathways that were recently found to be functionally associated with *EWS-FLI1* are also identified by our approach, such as chromatin remodeling (Riggi et al., 2014), splicing pathways (Selvanathan et al., 2015). Furthermore, some studies showed that *CRM1* (*XPO1*) inhibition can promote cytotoxicity in Ewing sarcoma cells by repressing *EWS-FLI1*-dependent *IGF-1* signaling (Sun et al., 2016) and *NR0B1* is required for the oncogenic phenotype mediated by *EWS-FLI1* in Ewing's sarcoma (Kinsey et al., 2006; García-Aragoncillo et al., 2008). Our approach also predicted *CRM1* and *NR0B1* are among top 5% genes that are functionally associated with *EWS-FLI1*.

**PREDICTION EVALUATION USING LITERATURE-BASED BENCHMARK GENE SETS AND** **DATA-DRIVEN GENE SIGNATURES ASSOCIATED WITH *EWS-FLI1* OR EWING’S SARCOMA**

We collected several literature-based benchmark gene sets to comprehensively evaluate our prediction using the ROC analysis, such as *EWS-FLI1* related genes (Fig 4A in the main paper). We also applied two other methods evaluate our predictions: GSEA and the Mann-Whitney-Wilcoxon test. The following table lists p-values of all evaluations using the two methods (please note that the minimal p.value of fGSEA output is 0.0001. So, pval =0.0001 indicates the real p.value<=0.0001).

| **Benchmarks** | **Wilcoxon.pval** | **GSEA.pval** |
| --- | --- | --- |
| Ewing_Genes | 1.3615e-36 | 0.0001 |
| EWS_FLI1_Genes | 3.5138e-14 | 0.001 |
| CancerPathway_Genes | 9.2096e-119 | 0.001 |
| Drug_TargetGenes | 2.4374e-23 | 0.0001 |
| Drug_Screening | 4.9497e-43 | 0.0001 |

In addition, four data-driven gene signatures that are associated with *EWS-FLI1* or Ewing’s sarcoma were used to evaluate our prediction of *EWS-FLI1*. These genes sets include 33 genes that are significantly de-regulated in Ewing's sarcoma cell lines after knockdown of *EWS-FLT1* by RNAi (Siligan et al., 2005), 60 genes that significantly de-regulated in neuroblastoma cell lines after expressing *EWS-FLI1* fusion protein (Rorie et al., 2004), 1607 genes that are significantly differentially expressed in Ewing TC71 and EWS502 cells after knockdown of *EWS-FLT1* by RNAi (Kinsey et al., 2006), and 88 genes that are highly expressed in Ewing's famliy tumors and are also up-regulated in RD-EF cells after expressing *EWS-FLI1* (Hu-Lieskovan et al., 2005). The evaluation results indicate that our prediction of *EWS-FLI1* correlates well with these gene signatures (S4 Fig).

**REFERENCES**

Anderson JL, Denny CT, Tap WD, Federman N. Pediatric sarcomas: translating molecular pathogenesis of disease to novel therapeutic possibilities. Pediatr Res. 2012;72(2):112-21.

Arvand A, Denny CT. Biology of EWS/ETS fusions in Ewing's family tumors. Oncogene. 2001;20:5747–54.

Benini S, Manara MC, Cerisano V, Perdichizzi S, Strammiello R, Serra M, et al. Contribution of MEK/MAPK and PI3-K signaling pathway to the malignant behavior of Ewing’s sarcoma cells: therapeutic prospects. Int. J. Cancer. 2004;108:358–66.

Bilke S, Schwentner R, Yang F, Kauer M, Jug G, Walker RL, et al. Oncogenic ETS fusions deregulate E2F3 target genes in Ewing sarcoma and prostate cancer. Genome Res. 2013;23(11):1797-809.

Chandhanayingyong C, Kim Y, Staples JR, Hahn C, Lee FY. MAPK/ERK Signaling in Osteosarcomas, Ewing Sarcomas and Chondrosarcomas: Therapeutic Implications and Future Directions. 2012;Sarcoma 2012: 404810.

DuBois SG, Marina N, Glade-Bender J. Angiogenesis and vascular targeting in Ewing sarcoma: a review of preclinical and clinical data. 2010;Cancer 116(3): 749-57.

García-Aragoncillo E, Carrillo J, Lalli E, Agra N, Gómez-López G, Pestaña A, et al. DAX1, a direct target of EWS/FLI1 oncoprotein, is a principal regulator of cell-cycle progression in Ewing's tumor cells. Oncogene. 2008;27(46):6034-43.

Griffith M, Griffith OL, Coffman AC, Weible JV, McMichael JF, Spies NC, et al. DGIdb: mining the druggable genome. Nat Methods 2013;10(12):1209-10.

Hodge AE, Altman RB, Klein TE. The PharmGKB: integration, aggregation, and annotation of pharmacogenomic data and knowledge. Clin Pharmacol Ther. 2007;81(1):21-4.

Hu-Lieskovan S, Zhang J, Wu L, Shimada H, Schofield DE, Triche TJ. EWS-FLI1 fusion protein up-regulates critical genes in neural crest development and is responsible for the observed phenotype of Ewing's family of tumors. Cancer Res. 2005;65(11): 4633-44.

Janknecht R. EWS-ETS oncoproteins: the linchpins of Ewing tumors. Gene. 2005;363:1-14.

Jully B, Rajkumar T. Potential molecular targets for Ewing's sarcoma therapy. Indian J Med Paediatr Oncol. 2012;33(4):195-202.

Kelleher FC, Thomas DM. Molecular pathogenesis and targeted therapeutics in Ewing sarcoma/primitive neuroectodermal tumours. Clin Sarcoma Res. 2012;2(1):6.

Kinsey M, Smith R, Lessnick SL. NR0B1 is required for the oncogenic phenotype mediated by EWS/FLI in Ewing's sarcoma. Mol Cancer Res. 2006;4(11):851-9.

Knox C, Law V, Jewison T, Liu P, Ly S, Frolkis A, et al. DrugBank 3.0: a comprehensive resource for 'omics' research on drugs. Nucleic Acids Res 2011;39(Database issue):D1035-41.

Kovar H. Downstream EWS/FLI1 - upstream Ewing's sarcoma. Genome Med. 2010;2(1):8.

Lissat A, Chao MM, Kontny U. Targeted Therapy in Ewing Sarcoma. ISRN Oncol. 2012: 609439.

Martín Liberal J, Lagares-Tena L, Sáinz-Jaspeado M, Mateo-Lozano S, García Del Muro X, Tirado OM. Targeted therapies in sarcomas: challenging the challenge. Sarcoma. 2012:626094.

Navarro D, Agra N, Pestaña A, Alonso J, González-Sancho JM. The EWS/FLI1 oncogenic protein inhibits expression of the Wnt inhibitor DICKKOPF-1 gene and antagonizes beta-catenin/TCF-mediated transcription. Carcinogenesis. 2010;31(3):394-401.

Ordóñez JL, Osuna D, Herrero D, de Alava E, Madoz-Gúrpide J. Advances in Ewing's sarcoma research: where are we now and what lies ahead? Cancer Res. 2009;69(18):7140-50.

Prieur A, Tirode F, Cohen P, Delattre O. EWS/FLI-1 silencing and gene profiling of Ewing cells reveal downstream oncogenic pathways and a crucial role for repression of insulin-like growth factor binding protein 3. Mol Cell Biol. 2004;24(16):7275-83.

Riggi N, Knoechel B, Gillespie SM, Rheinbay E, Boulay G, Suvà ML, et al. EWS-FLI1 utilizes divergent chromatin remodeling mechanisms to directly activate or repress enhancer elements in Ewing sarcoma. Cancer Cell. 2014;26(5):668-81.

Rorie CJ, Thomas VD, Chen P, Pierce HH, O'Bryan JP, Weissman BE. The Ews/Fli-1 fusion gene switches the differentiation program of neuroblastomas to Ewing sarcoma/peripheral primitive neuroectodermal tumors. Cancer Res. 2004;64(4):1266-77.

Selvanathan SP, Graham GT, Erkizan HV, Dirksen U, Natarajan TG, Dakic A, et al. Oncogenic fusion protein EWS-FLI1 is a network hub that regulates alternative splicing. Proc Natl Acad Sci. 2015;112(11):E1307-16.

Siligan C, Ban J, Bachmaier R, Spahn L, Kreppel M, Schaefer KL, et al. EWS-FLI1 target genes recovered from Ewing's sarcoma chromatin. Oncogene. 2005;24(15):2512-24.

Subbiah V, Anderson P. Targeted Therapy of Ewing's Sarcoma. Sarcoma 2011: 686985.

Sun H, Lin DC, Cao Q, Guo X, Marijon H, Zhao Z, et al. CRM1 Inhibition Promotes Cytotoxicity in Ewing Sarcoma Cells by Repressing EWS-FLI1-Dependent IGF-1 Signaling. Cancer Res. 2016;76(9):2687-97.

Uren A, Merchant MS, Sun CJ, Vitolo MI, Sun Y, Tsokos M, et al. Beta-platelet-derived growth factor receptor mediates motility and growth of Ewing's sarcoma cells. Oncogene. 2003;22(15):2334-42.

Zhu F, Han B, Kumar P, Liu X, Ma X, Wei X, et al. (2010) Update of TTD: Therapeutic target database. Nucleic Acids Res 38(Database issue): D787-791.
