# Supplementary material for: FusionPathway: Prediction of pathways and therapeutic targets associated with gene fusions in cancer
Source: PLoS Comput Biol. 2018 Jul 24;14(7):e1006266. doi: 10.1371/journal.pcbi.1006266 (PMC6075785; doi:10.1371/journal.pcbi.1006266)
Supplement: S4 Text — (DOCX) [file pcbi.1006266.s004.docx]

**Supporting Information For The FUS-DDIT3 Prediction**

**INTRODUCTION**

Myxoid liposarcoma is the second most common subtype of liposarcoma, which is characterized by translocation of chromosomes 12 and 16 (t12;16)(q13;p11) resulting in a fusion gene between *FUS* and *DDIT3* that is present in over 95% of cases (Conyers et al., 2011; Guan et al., 2015). The corresponding chimeric protein retains the RNA binding and activation domain of the *FUS* protein and DNA-binding domain of the *DDIT3* protein (Willems et al., 2010). *FUS-DDIT3* plays a specific and critical role in the pathogenesis of Myxoid liposarcoma (Rodriguez et al., 2013). The *FUS-DDIT3* fusion is thought to interfere with normal adipocytic differentiation through the *CEBP* transcription factors and *PPARG* and are likely involved in activation of a number of oncogeneic pathways (Willems et al., 2010; Conyers et al., 2011; Guan et al., 2015). Currently, treatments for Myxoid liposarcoma consist of surgical resection, radiation therapy, and cytotoxic chemotherapy. Therefore, therapeutics strategies targeting the pathways associated with *FUS-DDIT3* may help improve clinical outcomes.

**PREDICTION OF *FUS-DDIT3* PATHWAYS**

The domain-based network approach was used to infer pathways that are functionally associated with *FUS-DDIT3*. GSEA association analysis revealed that several known *FUS-DDIT3* pathways are highly functionally associated with *FUS-DDIT3* in our prediction (S5 Fig), such as adipocytokine signaling (Pérez-Mancera et al., 2008), DNA damage (Rulten et al., 2014), *NF-kB* pathways (Willems et al., 2010), *PI3K/Akt* pathway (Demicco et al., 2012), and *FGFR* pathway (Künstlinger et al., 2015). We also analyzed the gene expression data of myxoid liposarcoma-derived cell lines upon *FUS-DDIT3* knockdown (Rodriguez et al., 2013) to examine the deregulation of pathways. The GSEA deregulation analyses also shows these pathways are significantly deregulated upon *FUS-DDIT3* knockdown (S5 Fig). Other cancer related pathways, such as development, cell cycle, apoptosis, *WNT*, and DNA repair pathways, are also predicted by our prediction (S5 Fig). Furthermore, we also used several types of benchmark gene sets to comprehensively evaluate our prediction (Fig 4B in the main text). The evaluation results indicate that our approach can successfully identify most known *FUS-DDIT3*-associated genes or pathways that may play a role in the oncogenesis of Myxoid liposarcoma.

**PREDICTION EVALUATION OF *FUS-DDIT3* USING LITERATURE-BASED BENCHMARK GENE SETS AND** **DATA-DRIVEN GENE SIGNATURES ASSOCIATED WITH TRABECTEDIN**

We collected several literature-based benchmark gene sets to comprehensively evaluate our prediction using the ROC analysis, such as *FUS-DDIT3* related genes (Fig 4B in the main paper). We also applied two other methods evaluate our predictions: GSEA and the Mann-Whitney-Wilcoxon test (more details in section S3.2). The following table lists p-values of all evaluations using the two methods (please note that the minimal p.value of fGSEA output is 0.0001. So, pval =0.0001 indicates the real p.value<=0.0001).

|  |  |  | |  |
| --- | --- | --- | --- | --- |
| **Benchmarks** | **Wilcoxon.pval** | | **GSEA.pval** | |
| Myxoid_Genes | 2.6925e-16 | | 0.0001 | |
| FUS_DDIT3_Genes | 1.1919e-06 | | 0.0001 | |
| CancerPathway_Genes | 3.4920e-87 | | 0.0001 | |
| Drug_Screening | 1.9094e-33 | | 0.0001 | |

In addition, Myxiod liposarcoma has been shown to be highly sensitive to Trabectedin due to the ability of the drug to block the transactivation of *FUS-DDIT3* (Forni et al., 2009; Gronchi et al., 2012). Therefore, four data-driven gene signatures that are associated with Trabectedin were used to evaluate our prediction of *FUS-DDIT3*. These genes sets include 86 genes that are significantly de-regulated by Trabectedin in HeLa cells (Gajate et al., 2002), 50 genes that are significantly down-regulated by Trabectedin and its synthetic analog phthalascidin Pt 650 in in HCT116 cells (Martinez et al., 2001), 342 genes that are significantly de-regulated in at least 8 of 11 sarcoma cell lines by Trabectedin (Martínez et al., 2005), and 70 genes that are significantly de-regulated in chondrosarcoma and ovarian carcinoma cell lines which developed resistance to trabectedin (Marchini et al., 2005). The evaluation results indicate that our prediction of *FUS-DDIT3* correlates well with these gene signatures (S6 Fig).

**REFERENCES**

Conyers R, Young S, Thomas DM. Liposarcoma: molecular genetics and therapeutics. Sarcoma. 2011;2011:483154.

Demicco EG, Torres KE, Ghadimi MP, Colombo C, Bolshakov S, Hoffman A, et al. Involvement of the PI3K/Akt pathway in myxoid/round cell liposarcoma. Mod Pathol. 2012;25(2):212-21.

Forni C, Minuzzo M, Virdis E, Tamborini E, Simone M, Tavecchio M, et al. Trabectedin (ET-743) promotes differentiation in myxoid liposarcoma tumors. Mol Cancer Ther. 2009;8(2):449-57.

Gajate C, An F, Mollinedo F. Differential cytostatic and apoptotic effects of ecteinascidin-743 in cancer cells. Transcription-dependent cell cycle arrest and transcription-independent JNK and mitochondrial mediated apoptosis. J Biol Chem. 2002;277(44):41580-9.

Gronchi A, Bui BN, Bonvalot S, Pilotti S, Ferrari S, Hohenberger P, et al. Phase II clinical trial of neoadjuvant trabectedin in patients with advanced localized myxoid liposarcoma. Ann Oncol. 2012;23(3):771-6.

Guan Z, Yu X, Wang H, Wang H, Zhang J, Li G, et al. Advances in the targeted therapy of liposarcoma. Onco Targets Ther. 2015;8:125-36.

Künstlinger H, Fassunke J, Schildhaus HU, Brors B, Heydt C, Ihle MA, et al. FGFR2 is overexpressed in myxoid liposarcoma and inhibition of FGFR signaling impairs tumor growth in vitro. Oncotarget. 2015;6(24):20215-30.

Marchini S, Marrazzo E, Bonomi R, Chiorino G, Zaffaroni M, Weissbach L, et al. Molecular characterisation of two human cancer cell lines selected in vitro for their chemotherapeutic drug resistance to ET-743. Eur J Cancer. 2005;41(2):323-33.

Martinez EJ, Corey EJ, Owa T. Antitumor activity- and gene expression-based profiling of ecteinascidin Et 743 and phthalascidin Pt 650. Chem Biol. 2001;8(12):1151-60.

Martínez N, Sánchez-Beato M, Carnero A, Moneo V, Tercero JC, Fernández I, et al. Transcriptional signature of Ecteinascidin 743 (Yondelis, Trabectedin) in human sarcoma cells explanted from chemo-naive patients. Mol Cancer Ther. 2005;4(5):814-23.

Pérez-Mancera PA, Bermejo-Rodríguez C, Sánchez-Martín M, Abollo-Jiménez F, Pintado B, Sánchez-García I. FUS-DDIT3 prevents the development of adipocytic precursors in liposarcoma by repressing PPARgamma and C/EBPalpha and activating eIF4E. PLoS One. 2008;3(7):e2569.

Rodriguez R, Tornin J, Suarez C, Astudillo A, Rubio R, Yauk C, et al. Expression of FUS-CHOP fusion protein in immortalized/transformed human mesenchymal stem cells drives mixoid liposarcoma formation. Stem Cells. 2013;31(10):2061-72.

Rulten SL, Rotheray A, Green RL, Grundy GJ, Moore DA, Gómez-Herreros F, et al. PARP-1 dependent recruitment of the amyotrophic lateral sclerosis-associated protein FUS/TLS to sites of oxidative DNA damage. Nucleic Acids Res. 2014;42(1):307-14.

Willems SM, Schrage YM, Bruijn IH, Szuhai K, Hogendoorn PC, Bovée JV. Kinome profiling of myxoid liposarcoma reveals NF-kappaB-pathway kinase activity and casein kinase II inhibition as a potential treatment option. Mol Cancer. 2010;9:257.
