# Supplementary figures and images for: FusionPathway: Prediction of pathways and therapeutic targets associated with gene fusions in cancer
Source: PLoS Comput Biol. 2018 Jul 24;14(7):e1006266. doi: 10.1371/journal.pcbi.1006266 (PMC6075785; doi:10.1371/journal.pcbi.1006266)

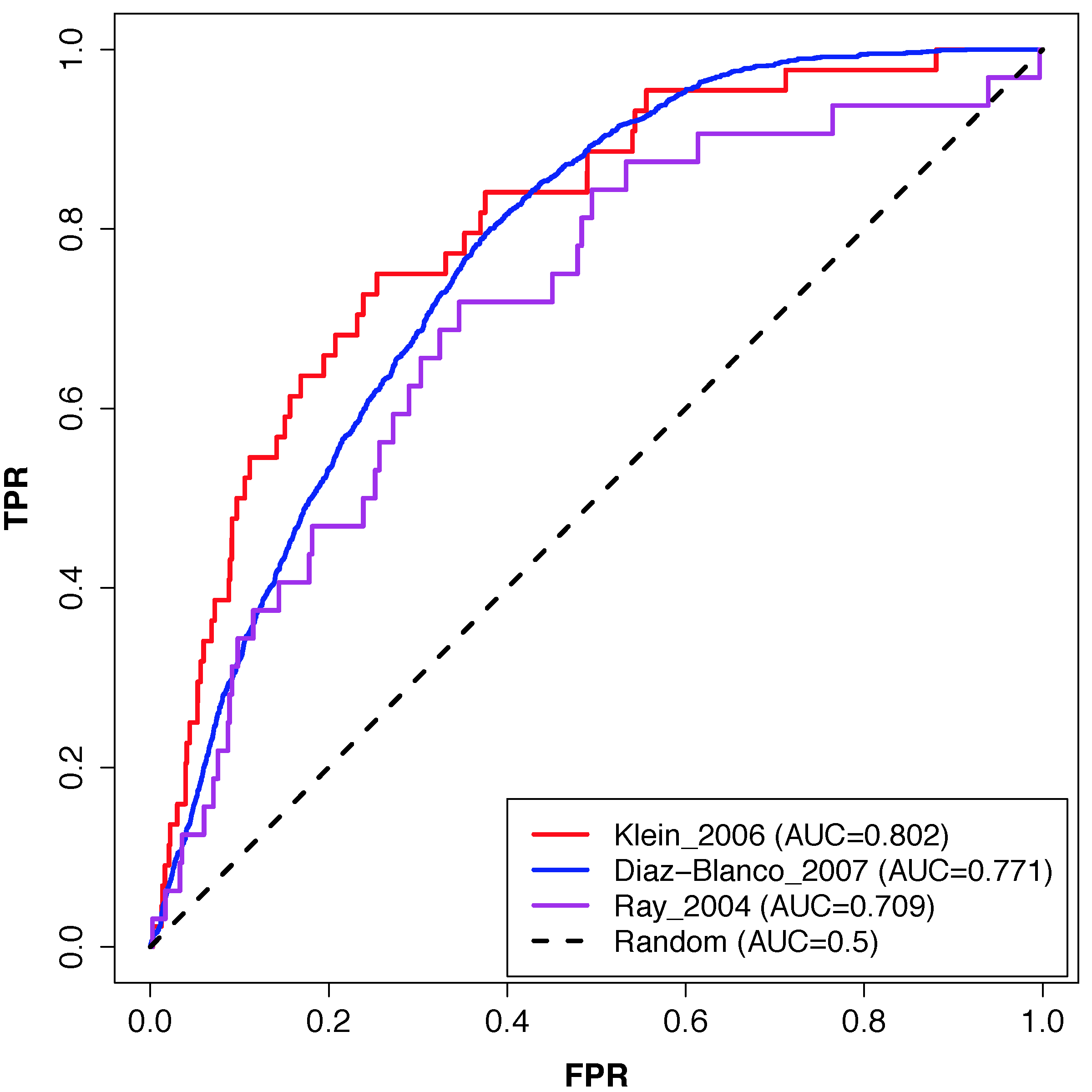

Supplement: S1 Fig — The three data-driven gene signatures were collected from several previous works (detailed in S2 Text). (TIFF) [file pcbi.1006266.s005.tiff]

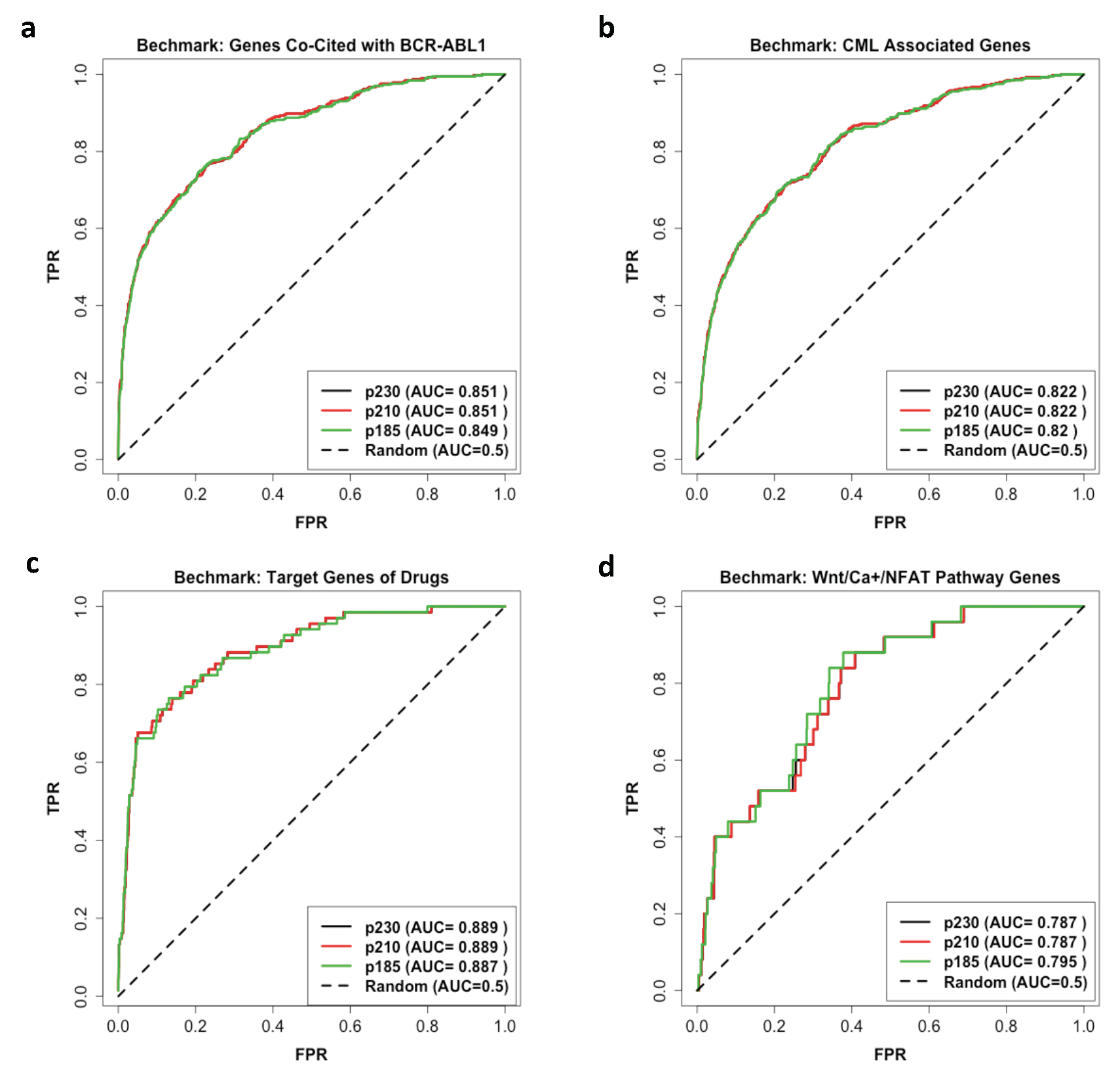

Supplement: S2 Fig — The three known BCR-ABL1 variants are denoted “p185”, “p210”, and “p230” respectively. (a) genes co-cited with BCR-ABL1 in literature. (b) CML associated genes. (c) target genes of drugs that have been already in clinical trials or used for treatment of CML (d) 26 Wnt/Ca+/NFAT pathway genes, which were identified by RNAi-based synthetic lethal screen with imatinib mesylate in CML cells. (TIFF) [file pcbi.1006266.s006.tiff]

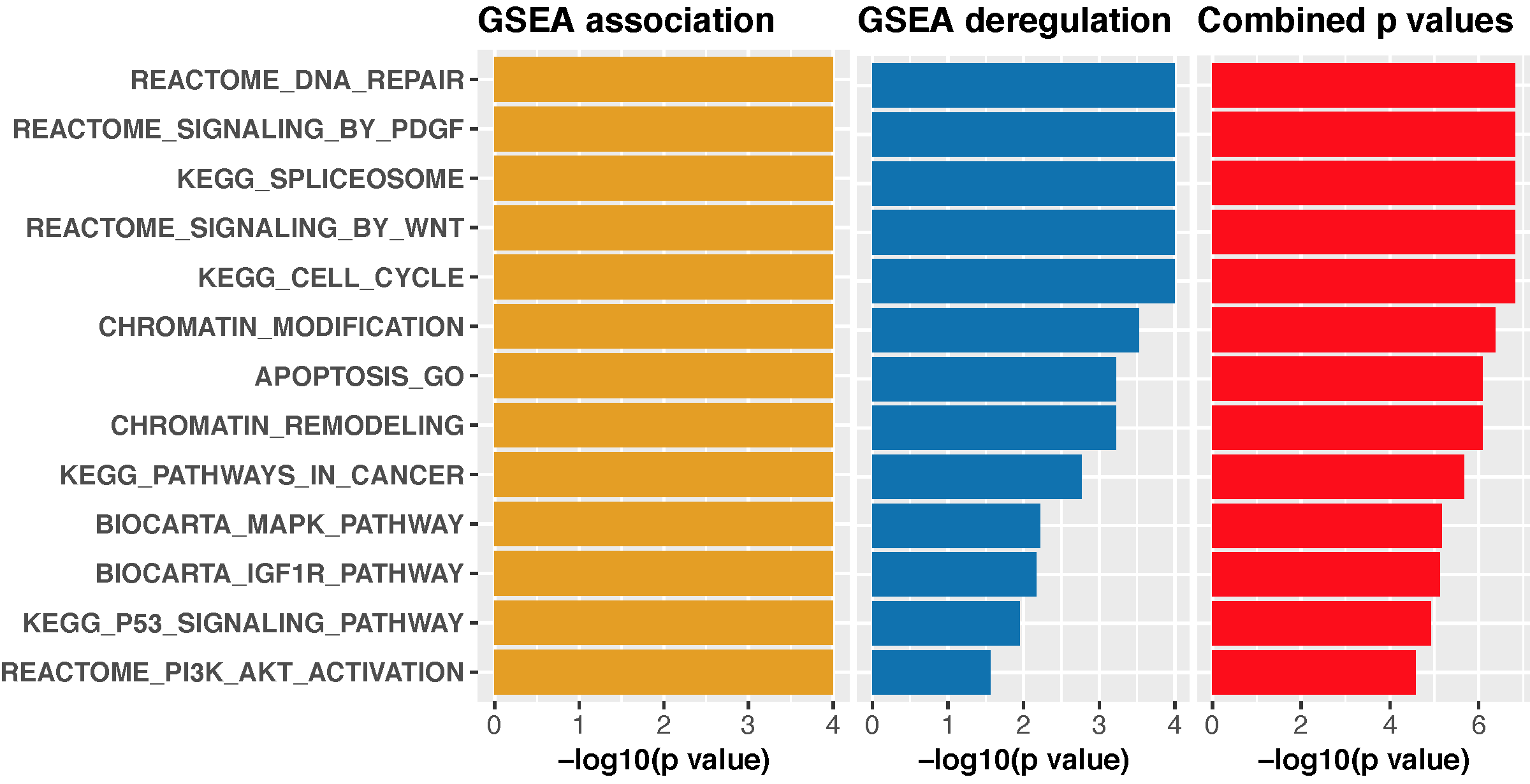

Supplement: S3 Fig — A bar in the graph represents the statistically significance of a given pathway in GSEA association analysis, GSEA deregulation analysis, or combination analysis using the truncated product method. The statistically significances are presented in the graph as–log10(p-value). (TIFF) [file pcbi.1006266.s007.tiff]

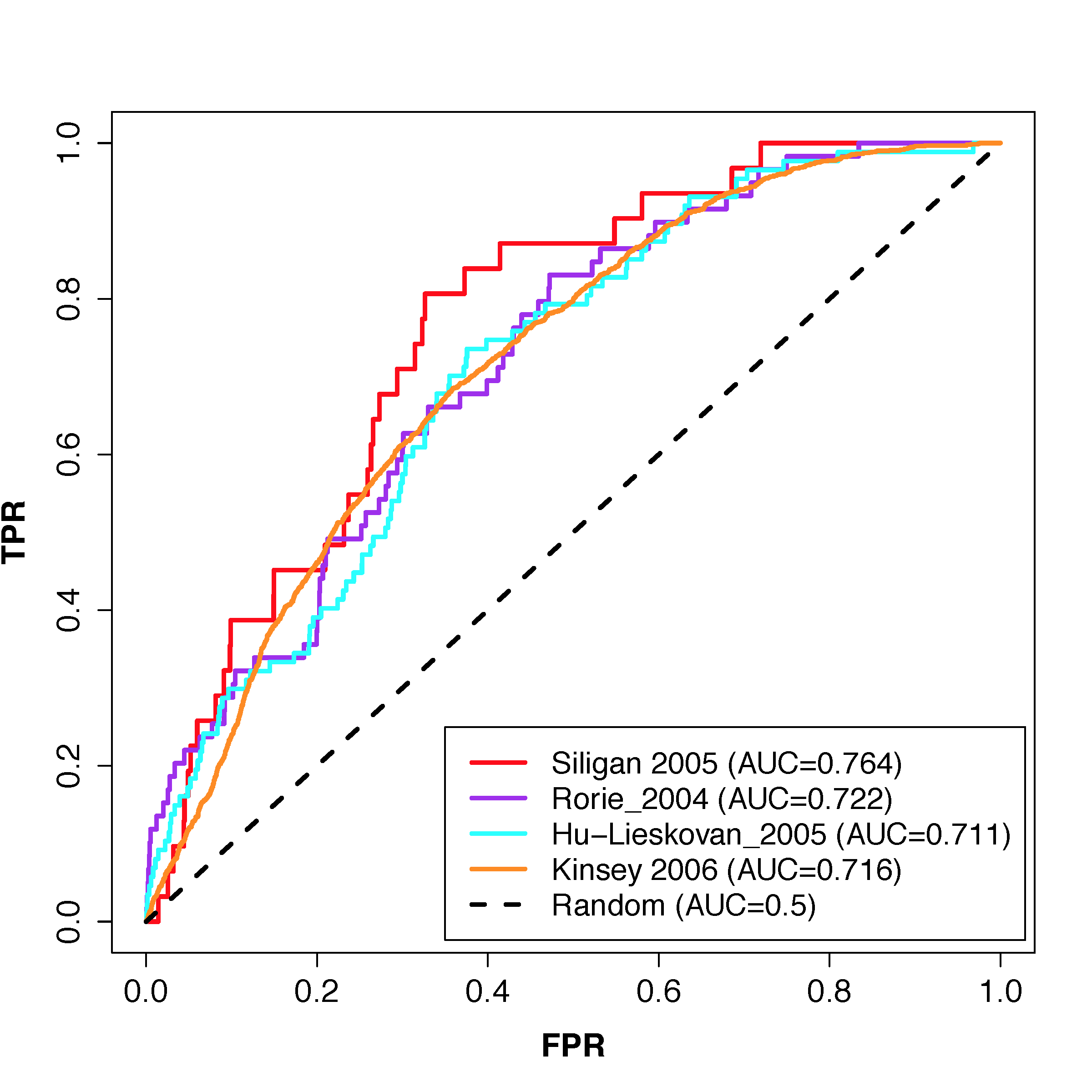

Supplement: S4 Fig — The four data-driven gene signatures were collected from several previous works (detailed in S3 Text). (TIFF) [file pcbi.1006266.s008.tiff]

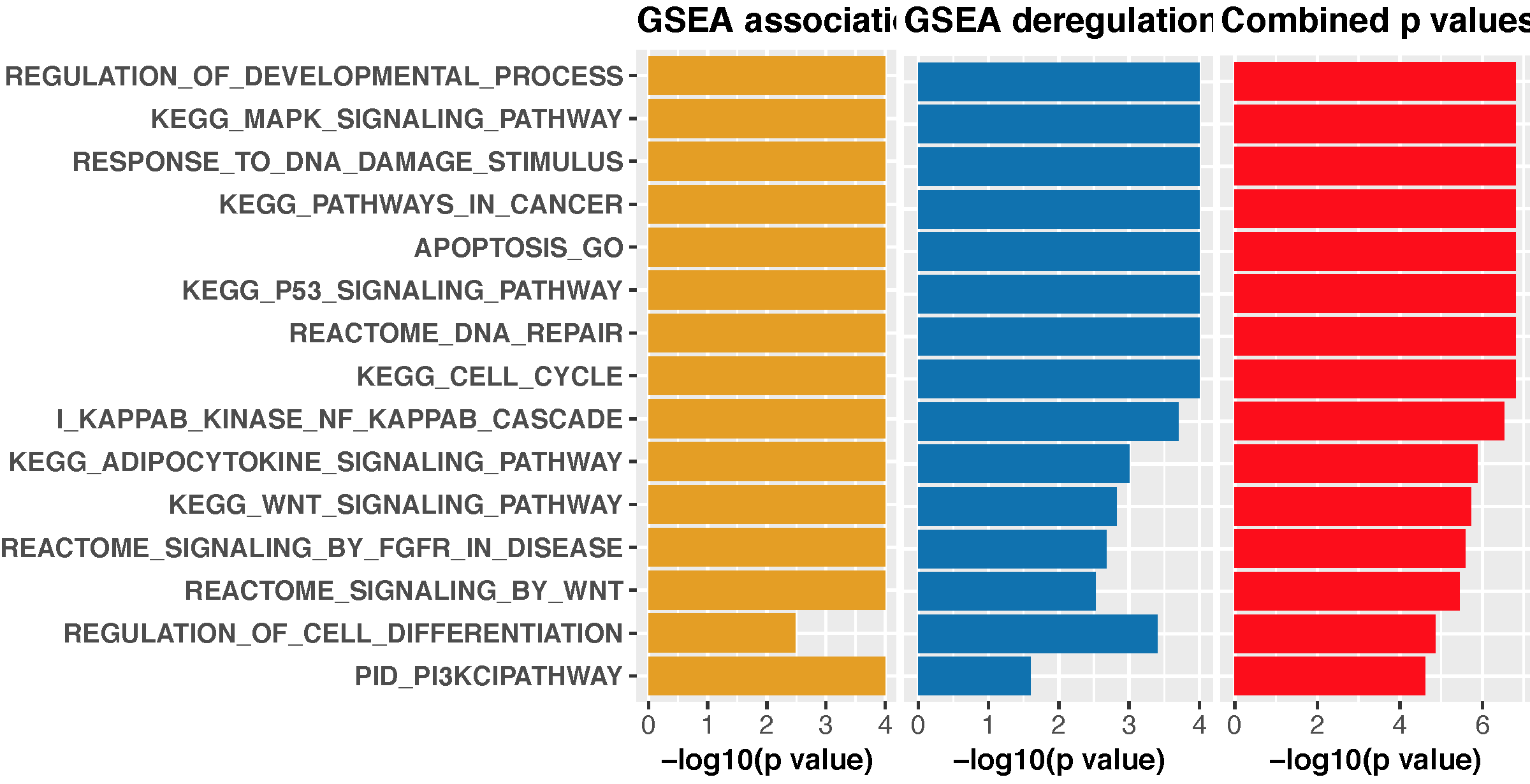

Supplement: S5 Fig — A bar in the graph represents the statistically significance of a given pathway in GSEA association analysis, GSEA deregulation analysis, or combination analysis using the truncated product method. The statistically significances are presented in the graph as–log10(p-value). (TIFF) [file pcbi.1006266.s009.tiff]

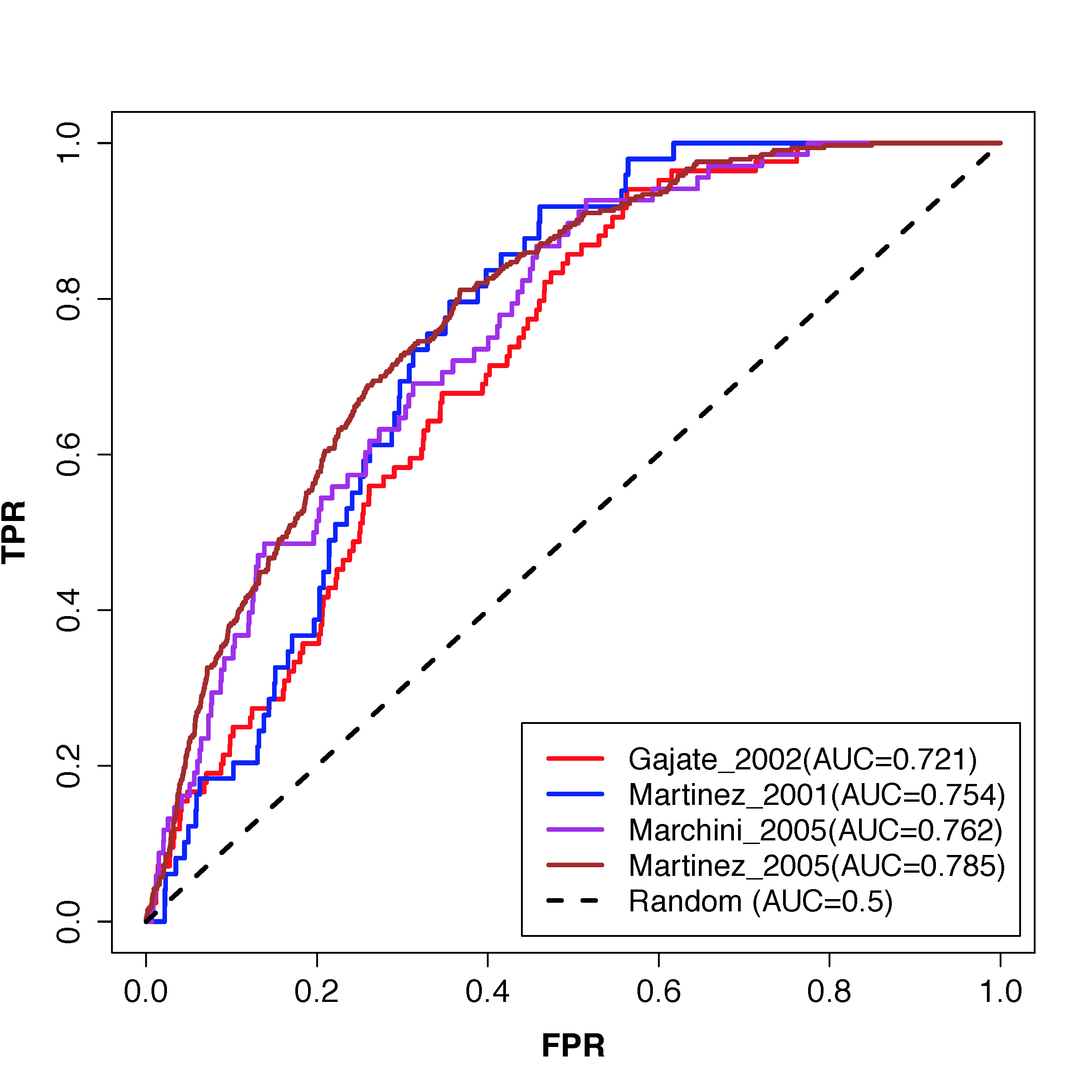

Supplement: S6 Fig — The four data-driven gene signatures associated with Trabectedin were collected from several previous works (detailed in S4 Text). (TIFF) [file pcbi.1006266.s010.tiff]
