# Supplementary material for: FusionPathway: Prediction of pathways and therapeutic targets associated with gene fusions in cancer
Source: PLoS Comput Biol. 2018 Jul 24;14(7):e1006266. doi: 10.1371/journal.pcbi.1006266 (PMC6075785; doi:10.1371/journal.pcbi.1006266)
Supplement: S1 Table — We complied 68 target genes of 24 compounds that have been already in clinical trials and used for treatment of CML from several review papers and drug databases (detailed in S2 Text). (DOCX) [file pcbi.1006266.s011.docx]

**S1 Table: Compounds that have been in clinical trials or used for treatment of CML**

| **Compounds** | **Target Genes** | **Developmental Status** |
| --- | --- | --- |
| Dasatinib | *SRC; ABL2; KIT; ABL1; FYN; LCK; PDGFRB; YES1;*  *STAT5B; EPHA2; EPHB1; EPHB2; EPHB3; EPHB4* | Approved |
| Nilotinib | *ABL1; PDGFRB; KIT; EPHB4* | Approved |
| Bosutinib | *SRC; HCK; BCR; ABL1; LYN; CDK2; MAP2K1; MAP2K2; MAP3K2; CAMK2G* | Approved |
| Bafetinib (INNO-406) | *ABL; PDGFRB; KIT; LYN* | Approved |
| Ponatinib (AP24534) | *SRC; KIT; BCR; ABL1; LYN; LCK; KDR; RET; FGFR1;*  *PDGFRA; TEK; FGFR2; FLT3; FGFR4; FGFR3* | Approved |
| Etoposide | *TOP2B; TOP2A* | Approved |
| Cytarabine | *POLB* | Approved |
| Homoharringtonine | *RPL3* | Approved |
| Cytarabine | *POLB* | Phase III |
| Saracatinib | *SRC* | Phase II |
| MK-0457 | *AURKA; AURKB; FLT3; JAK2; ABL* | Phase II |
| PHA-739358 | *AURKA; AURKB* | Phase II |
| Danusertib | *AURKA; AURKB; AURKC; NTRK1; FGFR1* | Phase II |
| Hydroxychloroquine | *TLR9; TLR7* | Phase II |
| Vincristine | *TUBA4A; TUBB* | Phase II |
| Valproic acid | *HDAC9; ABAT; ACADSB; OGDH; ALDH5A1; SCN1A; SCN10A; HDAC2; SCN11A; SCN2A; SCN3A; SCN4A* | Phase II |
| Rebastinib | *SRC; LYN; FGR; HCK; FLT3; TEK* | Phase I/ II |
| Everolimus | *MTOR* | Phase I/ II |
| Vatalanib | *FLT1; KDR; FLT4* | Phase I/ II |
| AT9283 | *AURKA; AURKB; AURKC; JAK2; JAK3* | Phase I/II |
| Vorinostat | *HDAC1; HDAC2; HDAC3; HDAC6; HDAC8* | Phase I |
| Tipifarnib | *FNTB* | Phase I |
| Zileuton | *ALOX5* | Phase I |
| HG-7-85-01 | *SRC; PDGFRA; PDGFRB; KDR; FLT3; RET; TEK; KIT; DDR1; BRAF* | Pre-clinical |
