# Supplementary material for: FusionPathway: Prediction of pathways and therapeutic targets associated with gene fusions in cancer
Source: PLoS Comput Biol. 2018 Jul 24;14(7):e1006266. doi: 10.1371/journal.pcbi.1006266 (PMC6075785; doi:10.1371/journal.pcbi.1006266)
Supplement: S2 Table — (DOCX) [file pcbi.1006266.s012.docx]

**S2 Table**: **Top 10% *BCR-ABL1*- associated genes that are significantly up-regulated upon Imatinib treatment**

| **Gene** | **log2 FC** | **Gene** | **log2 FC** |
| --- | --- | --- | --- |
| *BCL6* | 1.90 | *CSNK2A1* | 0.73 |
| *JAK2* | 1.56 | *SOS2* | 0.72 |
| *DDX58* | 1.40 | *NUMB* | 0.72 |
| *ZBTB20* | 1.18 | *SIRT2* | 0.71 |
| *RNF146* | 1.17 | *BPTF* | 0.70 |
| *CBLB* | 1.13 | *NCOA1* | 0.69 |
| *OPTN* | 1.05 | *ANKRD28* | 0.68 |
| *TNKS2* | 1.05 | *CREB1* | 0.67 |
| *STAT2* | 0.98 | *TSC22D4* | 0.64 |
| *ITPR2* | 0.97 | *ARHGAP35* | 0.64 |
| *CUL4B* | 0.93 | *USP9X* | 0.63 |
| *IFT20* | 0.88 | *ZBTB20* | 0.59 |
| *KLHL3* | 0.87 | *CSNK2A2* | 0.53 |
| *CUL4B* | 0.87 | *ARHGEF7* | 0.53 |
| *IGBP1* | 0.85 | *CEP70* | 0.52 |
| *TNRC6B* | 0.84 | *UBE2B* | 0.52 |
| *GAB1* | 0.82 | *RPS11* | 0.52 |
| *HNRNPA1* | 0.75 | *WIPI2* | 0.51 |
| *ATRX* | 0.75 | *NR2C2* | 0.50 |
| *PTPRC* | 0.74 | *ITPR2* | 0.50 |
