# Supplementary material for: FusionPathway: Prediction of pathways and therapeutic targets associated with gene fusions in cancer
Source: PLoS Comput Biol. 2018 Jul 24;14(7):e1006266. doi: 10.1371/journal.pcbi.1006266 (PMC6075785; doi:10.1371/journal.pcbi.1006266)
Supplement: S3 Table — We complied 67 target genes of 22 drugs that have been already in clinical trials and used for treatment of Ewing’s sarcoma from several review papers and drug databases (detailed in S3 Text). (DOCX) [file pcbi.1006266.s013.docx]

**S3 Table: Compounds that have been in clinical trials and used for treatment of Ewing’s sarcoma**

| **Compounds** | **Target Genes** | **Developmental Status** |
| --- | --- | --- |
| Doxorubicin | *AKR1C3; POR; CBR1; NOS2; NOS3; CBR3; NOS1; AKR1A1; NDUFS7; NQO1; TOP2A; XDH; NDUFS3; NDUFS2* | Approved |
| Rapamycin | *MTOR; FKBP1A; FGF2* | Phase I–III |
| Nutlin-3a | *MDM2* | Phase I–III |
| Imatinib | *CSF1R; PDGFRB; PDGFRA; KIT; PTGS1; NTRK1; ABL1; RET; DDR1* | Phase II |
| Alisertib (MLN8237) | *AURKA* | Phase II |
| Topotecan | *TOP1; TOP1MT* | Phase II |
| Cixutumumab | *IGFIR* | Phase I/II |
| Figitumumab | *IGFIR* | Phase I/II |
| Vismodegib | *SMO* | Phase I/II |
| Abexinostat | *HDAC1; HDAC3; HDAC4; HDAC2; HDAC9; HDAC7;*  *HDAC5; HDAC6; HDAC10; HDAC8* | Phase I/II |
| Vorinostat | *HDAC1; HDAC3; HDAC2; HDAC6; HDAC8* | Phase I/II |
| BIIB022 | *IGFIR* | Phase I |
| AVE-1642 | *IGFIR* | Phase I |
| OSI-906 | *IGFIR* | Phase I |
| XL-228 | *IGFIR; SRC; ABL1; ABL2* | Phase I |
| INSM-18 | *IGFIR; EGFR* | Phase I |
| MI-219 | *MDM2* | Phase I |
| Tenovins | *MDM2* | Phase I |
| Actinomycin D | *MDM2* | Phase I |
| Ridaforolimus | *MOTOR* | Phase I |
| Sorafenib | *FLT1; KIT; KDR; RET; PDGFRB; FLT3; FGFR1; FLT4;*  *RAF1; BRAF* | Phase I |
| Bevacizumab | *FCGR2A; VEGFA; C1QB; FCGR3B; FCGR2B; FCGR1A; C1QA; FCGR3A; C1QC; C1R; FCGR2C* | Phase I |
