# Supplementary material for: FusionPathway: Prediction of pathways and therapeutic targets associated with gene fusions in cancer
Source: PLoS Comput Biol. 2018 Jul 24;14(7):e1006266. doi: 10.1371/journal.pcbi.1006266 (PMC6075785; doi:10.1371/journal.pcbi.1006266)
Supplement: S4 Table — 76 sensitive compounds were identified in two Ewing’s sarcoma cell lines (TC32 and TC71) using the high-throughput screening assay. Target genes of these sensitive compounds were complied from available public databases, and 60 of the 76 compounds have known target genes. Totally, we have 197 drug targets of the 60 compounds. (DOCX) [file pcbi.1006266.s014.docx]

**S4 Table: Sensitive compounds for Ewing’s sarcoma cell lines and their target genes**

| **Compounds** | **Target Genes** |
| --- | --- |
| 17-AAG (Tanespimycin) | *HSP90AA1; HSP90AA2; HSP90AB1* |
| 17-DMAG (Alvespimycin) HCl | *HSP90AA1; HSP90AB1* |
| Alisertib (MLN8237) | *AURKA* |
| AMG-900 | *AURKA; AURKB; AURKC* |
| AT13387 | *HSP90AA1; HSP90AB1* |
| AT9283 | *ABL1; AURKA; AURKB; AURKC ; C14ORF129; JAK2; JAK3; MERTK; RET; RPS6KA3; TYK2; YES1* |
| AUY922 (NVP-AUY922) | *ALK; HSP90AA1; HSP90AA2; HSP90AB1* |
| AZD8055 | *AKT1S1; DEPTOR; HRAS; MAPKAP1; MLST8; MTOR; RICTOR; RPTOR* |
| Belinostat (PXD101) | *HDAC1* |
| BGT226 (NVP-BGT226) | *MTOR; PIK3CA* |
| BI 2536 | *PLK1* |
| BMN 673 | *ATM; ATR; BRCA1; BRCA2; PARP1; PARP2* |
| Cabazitaxel | *IGF2; TUBA4A; TUBB1* |
| Combretastatin A4 | *CDH1; TUBB1* |
| CUDC-907 | *PIK3C2A* |
| Doxorubicin hydrochloride | *CBR1; CYP2C19; FCGR2A; G6PD; GPX1; NOS2; NOS3; RAC2; SLC22A16; TOP2A* |
| Epirubicin hydrochloride | *CHD1; GSTP1; TOP2A; TOP2B* |
| Flavopiridol HCl | *CDK1; CDK2; CDK4; CDK5; CDK6; CDK7; CDK8; CDK9; EGFR; PYGM* |
| Floxuridine | *TYMS* |
| Ganetespib (STA-9090) | *ALK; HSP90AA1; HSP90AA2; HSP90AB1; UGT1A* |
| Gemcitabine hydrochloride | *BAZ2B; BTRC; CAMK4; CMPK1; DAPK1; DCP1B; ENOSF1; IL17F; KRAS; MAGEH1; MS4A2; MTHFR; PRB2; RRM1; RRM2; RRM2B; TYMS; UBASH3B; WEE1; WWOX; XRCC1; ZEB1* |
| GSK1070916 | *AURKA; AURKB; AURKC* |
| GSK2126458 (GSK458) | *AKT1; AKT2; AKT3; PIK3CA; PIK3CB; PIK3CD; PIK3CG; PIK3R1; PIK3R2; PIK3R3; PIK3R4; PIK3R5; PIK3R6* |
| GSK461364 | *PLK1* |
| Barasertib (AZD1152-HQPA) | *AURKB* |

**S4 Table: Sensitive compounds for Ewing’s sarcoma cell lines and their target genes (continued)**

| **Compounds** | **Target Genes** |
| --- | --- |
| Hesperadin | *MTTP; SOAT1; SOAT2* |
| Ispinesib (SB-715992) | *KLF11* |
| KX2-391 | *SRC* |
| INK 128 (MLN0128) | *CRTC1; CRTC2; MTOR* |
| Irinotecan | *ABCB1; ABCC1; ABCC2; ABCC5; ABCG1; ABCG2; BAIAP3; C18ORF56; C8ORF34; CYP3A4; CYP3A5; ENOSF1; ISG15; KLC1; PLCB1; PDZRN3; SLCO1B1; SLCO1B3; SEMA3C; SHMT1; TDP1; TOP1; TOP1MT; TYMS; VEGFA; XRCC3* |
| LAQ824 (Dacinostat) | *HDAC1; HDAC2; HDAC3; HDAC4; HDAC5; HDAC6; HDAC7; HDAC8; HDAC9; HDAC10* |
| Mitoxantrone | *ABCB1; ABCG2; GALNT14; MECP2; TOP2A; TOP2B* |
| MLN0905 | *PLK1* |
| MLN8054 | *AURKA* |
| MLN9708 | *PSMC2* |
| Obatoclax Mesylate (GX15-070) | *BCL2; BCL2L1; MCL1* |
| Oprozomib (ONX 0912) | *PSMB1; PSMB2; PSMB5; PSMD2* |
| Ouabain | *ATP1A1* |
| Panobinostat (LBH589) | *ABCB1; ATP4A; CYP2C19; HDAC1; HDAC2; HDAC3; HDAC4; HDAC5; HDAC6; HDAC7; HDAC8; HDAC9; HDAC10; HDAC11; SIRT1; SIRT2; SIRT3; SIRT4; SIRT5; SIRT6; SIRT7* |
| PIK-75 | *PIK3CA* |
| Rigosertib (ON-01910) | *PIK3CA; PIK3CB; PIK3CD; PLK1* |
| Pralatrexate | *DHFR; RFC1; TYMS* |
| R547 | *CDK2; CDK4; CDK7* |
| SB743921 | *KLF11* |
| SNS-314 Mesylate | *AURKA; AURKB; AURKC* |
| SN-38 | *HEATR7B1; TOP1; UGT1A1; UGT1A3; UGT1A4; UGT1A5; UGT1A6; UGT1A7; UGT1A8; UGT1A9; UGT1A10* |
| SNS-032 (BMS-387032) | *CDK2; CDK7; CDK9* |
| Torin 2 | *ATM; ATR; MTOR* |
| Trichostatin A (TSA) | *HDAC1; HDAC2; HDAC3; HDAC4; HDAC5; HDAC6; HDAC7; HDAC8; HDAC9* |
| Vinorelbine | *BRCA1; MTHFR; SMARCA4; TUBB; TUBB2A; XRCC1* |
| Volasertib (BI 6727) | *PLK1* |
| Nocodazole | *HPGDS* |

**S4 Table: Sensitive compounds for Ewing’s sarcoma cell lines and their target genes (continued)**

| **Compounds** | **Target Genes** |
| --- | --- |
| WYE-125132 (WYE-132) | *MTOR* |
| XL888 | *HSP90AA1; HSP90AB1* |
| YM155 (Sepantronium Bromide) | *BIRC5* |
| Zinc Pyrithione | *KCNQ1; KCNQ2; KCNQ4; KCNQ5* |
