# Supplementary material for: FusionPathway: Prediction of pathways and therapeutic targets associated with gene fusions in cancer
Source: PLoS Comput Biol. 2018 Jul 24;14(7):e1006266. doi: 10.1371/journal.pcbi.1006266 (PMC6075785; doi:10.1371/journal.pcbi.1006266)
Supplement: S5 Table — 48 sensitive compounds were identified in three Myxoid liposarcoma cell lines (MLS-1765-92, MLS-402-91, and MLS-DL221) using the high-throughput screening assay. Target genes of these sensitive compounds were complied from available public databases, and 38 of the 48 compounds have known target genes. Totally, we have 161 drug targets of the 38 compounds. (DOCX) [file pcbi.1006266.s015.docx]

**S5 Table: Sensitive compounds for Myxoid liposarcoma cell lines and their target genes**

| **Compounds** | **Target Genes** |
| --- | --- |
| 17-AAG (Tanespimycin) | *HSP90AA1; HSP90AA2; HSP90AB1* |
| 17-DMAG (Alvespimycin) HCl | *HSP90AA1; HSP90AB1* |
| Alisertib (MLN8237) | *AURKA* |
| AT13387 | *HSP90AA1; HSP90AB1* |
| AT9283 | *ABL1; AURKA; AURKB; AURKC; C14ORF129; JAK2; JAK3; MERTK; RET; RPS6KA3; TYK2; YES1* |
| AUY922 (NVP-AUY922) | *ALK; HSP90AA1; HSP90AA2; HSP90AB1* |
| BI 2536 | *PLK1* |
| Cabazitaxel | *IGF2; TUBA4A; TUBB1* |
| CUDC-907 | *PIK3C2A* |
| Dactinomycin | *CLCN6; MTHFR; MTR; TOP2A; TOP2B* |
| Dinaciclib (SCH727965) | *CCNE1; CDK1; CDK2; CDK5; CDK9; MYC* |
| Ganetespib (STA-9090) | *ALK; HSP90AA1; HSP90AA2; HSP90AB1; UGT1A* |
| Gemcitabine hydrochloride | *BAZ2B; BTRC; CAMK4; CMPK1; DAPK1; DCP1B; ENOSF1; IL17F; KRAS; MAGEH1; MS4A2; MTHFR; PRB2; RRM1; RRM2; RRM2B; TYMS; UBASH3B; WEE1; WWOX; XRCC1; ZEB1* |
| GSK1070916 | *AURKA; AURKB; AURKC* |
| GSK461364 | *PLK1* |
| Hesperadin | *MTTP; SOAT1; SOAT2* |
| Irinotecan | *ABCB1; ABCC1; ABCC2; ABCC5; ABCG1; ABCG2; BAIAP3; C18ORF56; C8ORF34; CYP3A4; CYP3A5; ENOSF1; ISG15; KLC1; PLCB1; PDZRN3; SLCO1B1; SLCO1B3; SEMA3C; SHMT1; TDP1; TOP1; TOP1MT; TYMS; VEGFA; XRCC3* |
| Ispinesib (SB-715992) | *KLF11* |
| LAQ824 (Dacinostat) | *HDAC1; HDAC2; HDAC3; HDAC4; HDAC5; HDAC6; HDAC7; HDAC8; HDAC9; HDAC10* |
| Mitoxantrone | *ABCB1; ABCG2; GALNT14; MECP2; TOP2A; TOP2B* |
| MLN0905 | *PLK1* |
| MLN9708 | *PSMC2* |
| Oprozomib (ONX 0912) | *PSMB1; PSMB2; PSMB5; PSMD2* |
| Ouabain | *ATP1A1* |
| Combretastatin A4 | *CDH1; TUBB1* |

**S5 Table: Sensitive compounds for Myxoid liposarcoma cell lines and their target genes (continued)**

| **Compounds** | **Target Genes** |
| --- | --- |
| Paclitaxel | *ABCB1; ABCC3; ABCC10; AURKA; BCL2; BIRC5; CYP1B1; CYP3A; CYP3A4; CYP3A5; DSCAM; EGFR; EIF4E2; ERCC2; ETS2; FCGR2A; GSTP1; KRAS; TLE3; MAP2; MAP4; MAPT; MTHFR; NR1I2; PDCD4; PGP; PTEN; STMN1; SYK; TIGD1; TIMP1; TLR4; TOP2A; TP53; TUBB; TUBB1; TUBB3* |
| Panobinostat (LBH589) | *ABCB1; ATP4A; CYP2C19; HDAC1; HDAC2; HDAC3; HDAC4; HDAC5; HDAC6; HDAC7; HDAC8; HDAC9; HDAC10; HDAC11; SIRT1; SIRT2; SIRT3; SIRT4; SIRT5; SIRT6; SIRT7* |
| PIK-75 | *PIK3CA* |
| Rigosertib (ON-01910) | *PIK3CA; PIK3CB; PIK3CD; PLK1* |
| SB743921 | *KLF11* |
| SNS-032 (BMS-387032) | *CDK2; CDK7; CDK9* |
| SNS-314 Mesylate | *AURKA; AURKB; AURKC* |
| Trichostatin A (TSA) | *HDAC1; HDAC2; HDAC3; HDAC4; HDAC5; HDAC6; HDAC7; HDAC8; HDAC9* |
| Vinorelbine | *BRCA1; MTHFR; SMARCA4; TUBB; TUBB2A; XRCC1* |
| Volasertib (BI 6727) | *PLK1* |
| XL888 | *HSP90AA1; HSP90AB1* |
| YM155 (Sepantronium Bromide) | *BIRC5* |
| SN-38 | *HEATR7B1; TOP1; UGT1A1; UGT1A3; UGT1A4; UGT1A5; UGT1A6; UGT1A7; UGT1A8; UGT1A9; UGT1A10* |
